# Quorum Sensing Inhibitory and Antifouling Activities of New Bromotyrosine Metabolites from the Polynesian Sponge *Pseudoceratina* n. sp.

**DOI:** 10.3390/md18050272

**Published:** 2020-05-21

**Authors:** Florent Tintillier, Céline Moriou, Sylvain Petek, Marilyne Fauchon, Claire Hellio, Denis Saulnier, Merrick Ekins, John N. A. Hooper, Ali Al-Mourabit, Cécile Debitus

**Affiliations:** 1 IRD, Univ de la Polynésie française, Ifremer, ILM, EIO, F-98713 Papeete, French Polynesia; florent.tintillier@gmail.com (F.T.); Cecile.Debitus@ird.fr (C.D.); 2 CNRS, Institut de Chimie des Substances Naturelles, Université Paris-Saclay, 91190 Gif-sur-Yvette, France; Celine.Moriou@cnrs.fr (C.M.); Ali.ALMOURABIt@cnrs.fr (A.A.-M.); 3 IRD, Univ Brest, CNRS, Ifremer, LEMAR, F-29280 Plouzane, France; marilyne.fauchon@univ-brest.fr (M.F.); claire.hellio@univ-brest.fr (C.H.); 4 Ifremer, IRD, ILM, Univ de la Polynésie française, EIO, F-98719 Taravao, French Polynesia; Denis.Saulnier@ifremer.fr; 5 Queensland Museum, PO Box 3300, South Brisbane BC 4101, Queensland, Australia; merrick.ekins@qm.qld.gov.au (M.E.); john.hooper@qm.qld.gov.au (J.N.A.H.)

**Keywords:** sponge, *Pseudoceratina*, quorum sensing inhibitory activity, antifouling, *Verongiida*, bromotyrosine

## Abstract

Four new brominated tyrosine metabolites, aplyzanzines C–F (**1**–**4**), were isolated from the French Polynesian sponge *Pseudoceratina* n. sp., along with the two known 2-aminoimidazolic derivatives, purealidin A (**5**) and **6,** previously isolated, respectively, from the sponges *Psammaplysilla purpurea* and *Verongula* sp. Their structures were assigned based on the interpretation of their NMR and HRMS data. The compounds exhibited quorum sensing inhibition (QSi) and antifouling activities against several strains of bacteria and microalgae. To our knowledge, the QSi activity of this type of bromotyrosine metabolite is described here for the first time.

## 1. Introduction

The unique chemotaxonomy, the tremendous molecular diversity, and the wide array of biological activities have made marine sponges of the order *Verongiida* a well-studied group. In addition to their bioactivities, the brominated tyrosine metabolites found in *Verongiida* are considered as chemotaxonomic markers [1,2,3]. Over 200 derivatives have been isolated so far; some of them produced by other orders, including *Agelasida* [4], *Dictyoceratida* [5], and *Tetractinellida* [6]. A recent study showed that the bacterium *Pseudovibrio denitrificans* Ab134, isolated from the sponge *Arenosclera brasiliensis* (order Haplosclerida), has the capacity to produce bromotyrosine derivatives [7]. These few counter-examples call into question the chemomarker status of these compounds. Setting aside misidentification of organisms and cross-contamination, this reminds us to remain cautious about the use of secondary metabolites for sponge taxonomy [8]. These bromotyrosine derivatives have very diverse structures, from the simplest such as subereaphenols [9], with two bromophenolic units such as psammaplysenes [10,11,12], amomoians [10,13,14], and aplyzanzines [14,15], to the most complex polycyclic such as archerines [16] or fistularins [17]. Among the most recently discovered structures, ma’edamines C and D are characterized by a tetrasubstituted pyridinium moiety, a novelty for this type of compound [18].

Over the course of evolution, benthic organisms in the marine environment have evolved many chemical strategies to interact with microorganisms, to protect themselves from pathogens, or to avoid being colonized [19,20,21]. In particular, sponges and their associated microbiota produce compounds that interfere with quorum sensing (QS) mechanisms [22,23,24,25,26] and protect themselves from biofouling [27,28]. In this context, bromotyrosines are known for their diverse biological activities [29,30], such as antimicrobial [31,32] and antifouling [33,34,35,36,37]. Some of these bioactivities also have the potential for use in human health as antiparasitic [38,39,40] or cytotoxic compounds [41,42].

Quorum sensing is a synchronization mechanism within a bacterial population. The bacteria use QS to communicate, regulating their behavior, and assess their population density. This is done through an auto-inducer (AI) permanently secreted into the medium. When the concentration of AI reaches a critical threshold, it generates a synchronized behavior such as luminescence, virulence, or aggregation to form biofilms [43]. Beyond the aspects of chemical ecology, this ability of marine organisms to interfere with QS could be used in the current context for the development of alternative antibacterial solutions to conventional antibiotics. Indeed, as this strategy does not rely on the eradication of bacteria, it can avoid resistance phenomena linked to selection pressure [44,45]. Furthermore, the strategy of marine organisms to prevent biofouling is to inhibit the first step, which is precisely the formation of bacterial biofilms that allow other organisms to attach [46].

**Figure 1:**
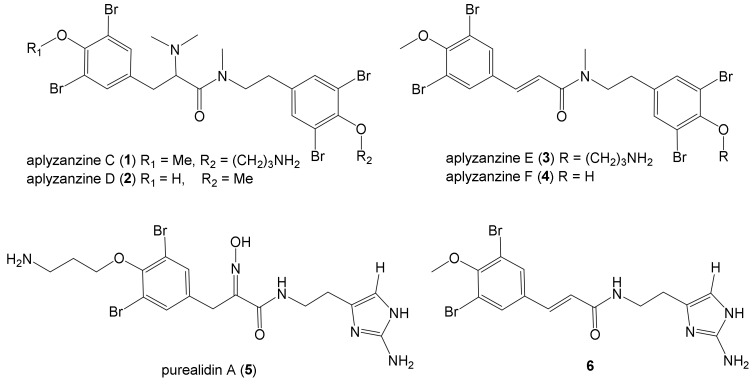
Structures of the isolated bromotyrosine metabolites.

As part of our survey of French Polynesian sponges [47,48,49] for new bioactive natural compounds in both health and sustainable aquaculture fields [50,51,52], we studied the marine sponge *Pseudoceratina* sp. 2081 and isolated four new tetrabromotyrosine derivatives exhibiting antifouling and quorum sensing inhibition (QSi) properties. We report here the isolation process, structure determination, and biological evaluation of these compounds, aplyzanzines C–F (**1**–**4**), along with two known molecules isolated there, purealidin A (**5**) and **6** (Figure 1). 

Aplyzanzine A, previously isolated from *Aplysina* sp. [15], is known for its antibacterial and antifungal activities [53], while aplyzanzine B and other close structures, such as anomoians and psammaplysenes, are mildly cytotoxic against a panel of different cancer cell lines [10,14]. Psammaplysene D, also isolated from a French Polynesian *Verongiidae,* displayed a substantial inhibition of acetylcholinesterase, probably as an anti-predatory defense through fish antifeedant activity [10].

## 2. Results and Discussion

### 2.1. Isolation and Structure Elucidation

A preliminary screening of biological activities performed on the ASE (accelerated solvent extraction) CH_2_Cl_2_/MeOH (1/1) crude extract of *Pseudoceratina* sp. 2081 sponge, revealed cytotoxic activity against the KB tumor cell lines (100% at 10 µg/mL). It also revealed quorum sensing inhibition of *Vibrio harveyi* with a 13/16 mm diameter of inhibition on the agar plate bioassay, inhibition of the mussel phenoloxydase activity (IC_50_ = 36.5 µg/mL), growth inhibition of the marine bacteria *Vibrio aestuarianus, Roseobacter littoralis,* and *Halomonas aquamarina* (MIC = 1 µg/mL), and of barnacle settlement (EC_50_ = 47.9 µg/mL). 

An aq-alcoholic extraction of the freeze-dried sponge was performed. The crude extract obtained was then partitioned between CH_2_Cl_2_ and water to give an organic soluble extract C, a precipitate P, and an aqueous layer. The aqueous layer was further partitioned against n-butanol to furnish the crude butanolic extract B. Bioguided fractionation of the extracts and the precipitate, using normal-phase and/or reversed-phase chromatography led to the isolation of compounds **1**, **3**, and **6** from B and P, **5** from B, and **2**, **4** from C.

**Figure 2:**
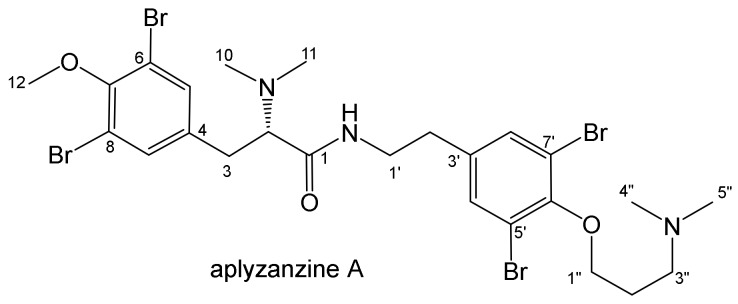
Structure of aplyzanzine A.

Aplyzanzine C (**1**) was obtained as a colorless varnish. Its HRESIMS data showed a [M + H]^+^ ion cluster at *m/z* 726, 728, 730, 732, and 734 in a 1:3:5:3:1 ratio, indicating the presence of four bromine atoms in the molecule. Analysis of the [M + H]^+^ peak at *m/z* 729.9170 suggested a C_24_H_31_N_3_O_3_Br_4_ formula for **1**, indicating nine degrees of unsaturation. The ^1^H-NMR spectrum (CD_3_OD, Table 1) showed signals concerning four aromatic protons between δ_H_ 7.45 and 7.55, and three methoxyl protons at *δ*_H_ 3.84/3.79, all of these doubled in a ~3:1 ratio (only the predominant signals are mentioned in the table). The ^13^C NMR spectrum revealed the presence of only one carboxyl at δ_C_ 168.3, and eight signals between δ_C_ 119.1 and 155.7, similar to those reported for aplyzanzine A [15]. The analysis of the 2D NMR experiments, including ^1^H-^1^H COSY, ^1^H-^13^C HSQC, and HMBC, allowed us to confirm the similarities with the main structure of aplyzanzine A (Figure 2), with a dibromo-methoxytyrosine part, a *para*-N-ethyl-dibromophenol moiety linked to a propylamine group. 

**Figure 3:**
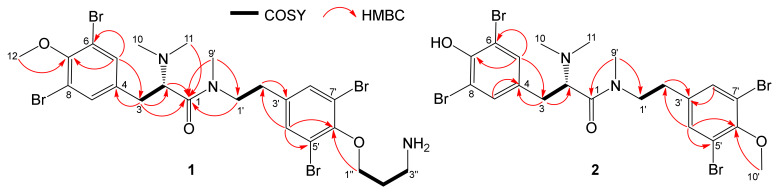
COSY and key HMBC correlations for aplyzanzines C (**1**) and D (**2**).

^1^H and ^13^C chemical shifts of the CH-2 at δ_H_ 4.65 (δ_C_ 66.3) suggested that compound **1** bears a dimethylamine group in accordance with the data reported for aplyzanzine A and similar compounds, such as aplyzanzine B and anomoian B [14], confirmed by HMBC correlation between CH_3_-10,11 at δ_H_ 2.91 (δ_C_ 42.7) and the carbonyl C-1. Finally, HMBC correlations observed between CH_3_-9’ at δ_H_ 2.65 (δ_C_ 35.8), C-1, and CH_2_-1’ at δ_H_ 3.47 (δ_C_ 50.5) confirm the position of this methyl on the amide nitrogen, leading to the structure assigned for aplyzanzine C (**1**) shown in Figure 3.

NMR spectra of aplyzanzine C (**1**) and the other compounds displayed doubled signals or complex multiplets. As previously reported in the literature, the NMR spectra of this family of compounds are complicated by the multiplicity of signals due to the presence of two rotational isomers trans/cis along the tertiary amide bond [11]. This behavior has been noted for tetrabromotyrosine derivatives such as anomians C–F and psammaplysenes F–I as well [10]. In Table 1 and Table 2, unless otherwise specified, the values shown are those of the predominant rotamer signals. The minor signals are often difficult to point to. Aplyzanzine D (**2**) was isolated as a colorless varnish. The HRESIMS data showed the [M + H]^+^ ion as a cluster at *m/z* 669, 671, 673, 675, and 677 in a 1:4:6:4:1 ratio, indicating the presence of four bromine atoms in the molecule. Analysis of the [M + H]^+^ peak at *m/z* 672.8610 suggested the molecular formula C_21_H_24_N_2_O_3_Br_4_ for compound **2**, indicating nine degrees of unsaturation. When compared to aplyzanzine C (**1**), the molecular formula lacked 57 mu corresponding to a C_3_H_7_N fragment. Examination of the 1D and 2D NMR spectra (Table 1) allowed the assignment of a similar basic structure as for **1**. NMR spectra differences of compounds **1** and **2** lie on the phenol substitution: compound **2** bears a hydroxyl on C-7 and a methoxyl group on C-6’, with CH_3_-10’ at δ_H_ 3.83 (δ_C_ 61.2). The structure **2** was thus assigned to aplyzanzine D.

Aplyzanzines C (**1**) and D (**2**) exhibited similar optical rotation values of respectively +26° and +27.4°. Comparison with similar compounds containing an l-tyrosine residue previously reported in the literature, such as suberedamines A (${\left[\alpha \right]}_{D}^{25}$ +21°) and B (${\left[\alpha \right]}_{D}^{25}$ +16°) [42], anomoian B (${\left[\alpha \right]}_{D}$ +10.5°), and aplyzanzine B (${\left[\alpha \right]}_{D}$ +47.5°) [14], indicates that our aplyzanzines C and D probably also contain l-tyrosine, with the same *S* configuration for C-2. It is obvious that we must carefully consider the comparison of these ${\left[\alpha \right]}_{D}$ values.

**Figure 4:**
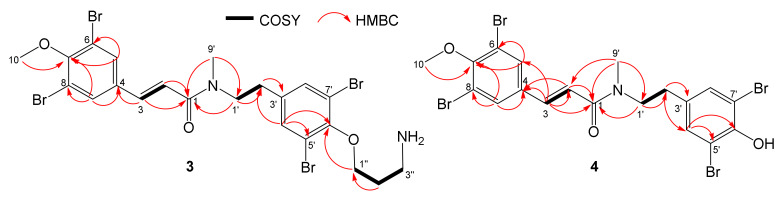
COSY and key HMBC correlations for aplyzanzines E (**3**) and F (**4**).

Aplyzanzine E (**3**) is a minor colorless compound obtained from both the precipitate and the butanolic phases. The HRESIMS data showed the [M + H]^+^ ion as a cluster at *m/z* 681, 683, 685, 687, and 689 mu, indicating the presence of four bromine atoms in the molecule again. Analysis of the [M + H]^+^ peak at *m/z* 684.8616 suggested the molecular formula C_22_H_24_N_2_O_3_Br_4_ for this compound, indicating ten degrees of unsaturation. The difference of molecular formulae with aplyzanzine C (**1**) suggested the loss of the dimethylamine fragment. The same dibromophenolic moieties found in aplyzanzine C (**1**) were observed on the 1D and 2D NMR spectra (Table 2). This was confirmed by the lack of the signals of N(CH_3_)_2_ at δ_H_ 2.91 (δ_C_ 42.7) on the ^1^H NMR spectrum of compound **3** but present on the spectrum of compound **1**. Regarding the UV spectra of compounds **1–3**, aplyzanzines C (**1**) and D (**2**) exhibited the same UV spectra with a maximum of absorption at 207 nm (in MeOH), but the spectrum of aplyzanzine E (**3**) displayed two maxima at 207 and 284 nm, suggesting the presence of additional π system confirmed by NMR signals of two olefinic protons at δ_H_ 6.96 (δ_C_ 138.4) and δ_H_ 6.60 (δ_C_ 120.7). Their coupling constant of 15 Hz is characteristic of a *trans* configuration of the double bond between CH-2 and CH-3. HMBC correlations of these two protons with the aromatic C-4 at δ_C_ 133.3 and the amidic carbonyl C-1 at δ_C_ 168.9 allowed to assign the position of this double bond between these two fragments. These data are consistent with those reported for psammaplysenes [10,11,12], which also contain a CH-2-CH-3 double bond. All these observations led us to assign the structure **3** to aplyzanzine E (Figure 4).

Aplyzanzine F (**4**), a minor component, was isolated from extract C as a colorless varnish. The HRESIMS data displayed a [M + H]^+^ ion cluster at *m/z* 622, 624, 626, 628, and 630 mu, indicating the presence of four bromine atoms in the molecule again. Analysis of the [M + H]^+^ signal at *m/z* 627.8020 suggested the molecular formula of **4** as C_19_H_17_NO_3_Br_4_, indicating ten degrees of unsaturation. Examination of the 1D and 2D NMR spectra, similar to those of aplyzanzine E (**3**) (Table 2), showed the presence of two rotamers in a 70/30 ratio. Analysis of the data of the predominant form allowed us to find the same moieties and connections as for aplyzanzine E (**3**) but lacking the propylamine fragment. This is consistent with the loss of 57 mu of the [M + H]^+^ ion, corresponding to a fragment C_3_H_7_N. The UV absorption spectra are also similar to the same maxima at 208 and 285 nm. The ^1^H NMR spectrum also displayed a coupling constant of 16Hz between the two olefinic CH at δ_H_ 6.97 (δ_C_ 138.8) and δ_H_ 6.64 (δ_C_ 120.7) and thus confirmed the *trans* configuration of this double bond.

The known natural products purealidin A (**5**) [54] and **6** [55] isolated from *Psammaplysilla purpurea* and *Verongula* sp. respectively, were identified by comparison of the NMR and MS data (Figures S22–S27, Tables S1–S2) with those reported in the literature [54,55,56].

Presently, 91 bromotyrosine derivatives have been described in the literature from sponges of the genus *Pseudoceratina* (ex-*Psammaplysilla*) [57]. Our compounds display similarities with some of them. The main skeleton of aplyzanzines C–F is close to aplysamine 4 [58], but differs by the presence of a dimethylamine group instead of the oxime on C-2, and also a methylated tertiary amide in aplyzanzines C (**1**) and D (**2**). Furthermore, the loss of the amine group in C-2 of aplyzanzines E (**3**) and F (**4**) leads to the double bond C-2-C-3. Aplyzanzines C (**1**) and D (**2**) are closer to aplyzanzine A [15] than any other described bromotyrosines, differing just by the methylation of the amidic nitrogen and the terminal amine group in C-3”. Aplyzanzines E (**3**) and F (**4**) also share similarities with psammaplysenes [10,11,12], in particular with the double bond C-2-C-3.

### 2.2. Biological Activities

#### 2.2.1. QSi General Screening

We have chosen the luminescence of a *V. harveyi* strain to evaluate the inhibitory activities of the QS. This luminescence is controlled by three AIs, activating 3 different QS pathways: *harveyi* auto inducer-1 (HAI-1), *cholerae* auto inducer-1 (CAI-1), and auto inducer-2 (AI-2) [59].

The aim of this screening was to find extracts and pure compounds inhibiting the luminescence without any antibiotic activity. Two bioassays were performed: a qualitative and quick test using disc diffusion on agar plates, followed by a bioassay in liquid media on 96-well plates, allowing the kinetic measurement of both bacterial growth and luminescence. This latter bioassay requires much smaller amounts of extracts and pure compounds. The crude MeOH/DCM (1:1) extract of *Pseudoceratina* sp. 2081 from Tuamotu, exhibited a 13/16 mm diameter of inhibition and no bactericidal activity, on the wild *V. harveyi* strain CIP 104774 and was thus selected for further investigation.

#### 2.2.2. Evaluation of the QS Inhibition of the Isolated Compounds

Compounds **1**, **3**, **6** were initially tested on solid medium, with 17, 0, and 21 mm of inhibition of luminescence respectively on the *Vibrio harveyi* strain CIP 104774, at 0.5 mg per 6 mm cellulose disk. This activity was further studied in the liquid media bioassay on the wild strain BB120 and three double mutants (Table 3). The mutants only expressed one of the three pathways controlling the luminescence by the auto inducers: JAF 375 (CAI-1 activated), JMH 597 (AI-2 activated), and JMH 612 (HAI-1 activated) [59,60,61]. Relative luminescence units found at the inflexion point of purified product and control (without any extract or compound) sigmoids were monitored in order to determine if one particular purified product accelerated or delayed luminescence when RLU reached 100,000.

None of the 6 purified products induced a significant change in the growth curve (compared to the control).

Aplyzanzine C (**1**) activated luminescence of the wild strain in liquid medium (13.4 ± 5.4 minutes) when used at the final concentration of 50 µg/mL only, whereas inhibiting luminescence on the agar plate bioassay. However, a slight inhibition of the luminescence of the 3 mutants at the lowest concentration was observed (16.7 ± 8.0 min. to 16.7 ± 0.1, depending on the strain used). 

Aplyzanzine D (**2**) and F (**4**) had relatively similar behavior, slightly QS activation on BB120 and JAF375 strains (from 7.9 ± 5.3 min. to 11.4 ± 3.9, depending purified compound and strain used), and weakly or not QSi on JMH597 and JMH612 strains.

Aplyzanzine E (**3**) and purealidin A (**5**) and (**6**) clearly inhibited QS of BB120 at 5 µg/mL, with luminescence delayed at 26.1 ± 5.7, 26.7 ± 0.5, and 44.1 ± 1.0 minutes, respectively. Aplyzanzine E (**3**) was inactive on agar plates but displayed a similar dose-dependent QSi on the 4 strains on the microwell bioassay. Since luminescence was affected in all three mutants, this may indicate that aplyzanzine E (**3**) acts downstream of Hfq cascade [60].

In the marine environment, it has been shown that many micro and macroorganisms have developed strategies to interfere with the QS of bacteria. These may be to auto-inducers’ enzymatic degradation, inhibition of their synthesis or inactivation of the receptors by binding of competing metabolites [62]. Only a few inhibitors have been clearly identified for bacteria of the genus *Vibrio.* Some inhibitors produced by bacteria include N-(2′-phenylethyl)-isobutyramide and 3-methyl-N-(2′-phenylethyl)-butyramide synthesized by *Halobacillus salinus* C42 [63] and 2-N-pentyl-4-quinolinol produced by *Alteromonas* sp. [64]. Isonaamidine A has been identified from the sponge *Leucetta chagosensis* [52] to affect the luminescence of *V. harveyi*, as do the halogenated furanones from the macroalgae *Delisea pulchra* [65] that also inhibit toxin production.

Less than thirty compounds, displaying a wide array of chemical structures, have been identified from sponges as QSi of different bacteria, such as sesterterpenes from *Luffariella variabilis* [66], furanosesterterpenes isolated from *Ircinia felix* [67], some brominated aminoimidazole derivatives, and brominated alkaloids [22,26,62,68]. In addition to QSi activity, some compounds also display antibiotic activity such as avarol and some alkaloids [22].

The QSi compounds **3**, **5,** and **6** share some chemical characteristics with previously identified QS inhibitors, such as the presence of several bromines and/or a phenol group. Purealidin A (**5**) and **6**, bear an aminoimidazolic moiety, like the QS inhibitors oroidin [68] or ageliferin [22]. However, to the best of our knowledge, QSi activity of this type of bromotyrosine derivative, with two dibromophenolic units or combining a dibromotyrosine part and an aminoimidazole moiety, is described here for the first time.

#### 2.2.3. Antifouling Activities

In addition to the general QSi screening (previously described in Section 2.1), an evaluation of antifouling activities was carried out on the same library of extracts. 

The purified compounds **1**–**6** were tested against 3 strains of microalgae and 3 strains of marine bacteria (Table 4) at concentrations up to 10 μg/mL to remain within a high activity range (presented in μM in the table).

Among the 6 compounds tested, the most active were **1**, **3** and **5**. Compound **1** is of great interest because it led to significant inhibition of both adhesion and growth of marine microalgae (with MIC values of 0.14 µM in all assays). Although its activity is lower than that of Seanine 211 [69], the fact that Seanine 211 is very toxic to the cells warrants the investigation of alternative molecules with a more specific and targeted activity. With this point of view, the combination of inhibiting adhesion and growth has a major advantage, allowing increased efficiency and reduced risk of the algae developing resistance. Thus compound **1** can be qualified as a promising compound with anti-microalgae activity. 

Compound **3** showed no activity towards microalgae and was specifically active towards marine bacteria. It led to the inhibition of adhesion of the two *Vibrio* strains studied (with MICs values of 0.15 µM) and to the inhibition of growth of *V. aestuarianus* (with MIC of 0.01 µM). 

Compound **5** is of great interest for antifouling applications, because it displays a broad spectrum of activity, along with targeted activities. Its antimicrobial activities are very close to Seanine 211 levels (with very low values of MICs), but unlike Seanine 211, the mode of action of compound **5** is not based on toxicity. It operates through a targeted mode of action on bacteria and microalgae, as it only affects adherence and not growth.

Compound **2** was less active than **1** and **3** and led to MICs values of 1.48 µM against growth of two microalgae and adhesion of one bacterial species, so it is not possible to consider this compound as a potential hit for the development of new antifouling solutions. Compounds **6** and **4** were active in a single test respectively: inhibiting the growth of *V. aestuarianus* (MIC = 0.22 µM) for compound **6**, and inhibiting the adhesion of *H. coffeaeformis* for compound **4** (MIC = 0.02 µM).

Furthermore, it is interesting to note that the QSi compounds **3** and **5**, are active against bacteria adhesion while **3** and **6** inhibit the growth of *V. aesturianus*.

More than one hundred thirty molecules from sponges have been described in the literature for their antifouling activities on diverse microorganisms or invertebrate larvae [57], including twenty-five natural bromotyrosine derivatives [6,33,34,70,71,72]. Concerning the genus *Pseudoceratina*, ceratinamides A and B, psammaplysins A and E, ceratinamine, moloka’iamine [70], and 5-bromoverongamine [33] exhibited different activities against micro and macro organisms involved in biofouling. 

Our compounds were tested on the same microorganisms as synoxazolidinones A and C, pulmonarin A and B [34], and ianthelline [6] (Table 4). Comparing their activities on microalgae, aplyzanzine C (**1**) is more effective in inhibiting their adhesion in general, while aplyzanzine F (**4**) inhibits specifically *H. coffeaeformis*. The growth inhibition of compound **1** is close to the activity of synoxazolidinone C, but is more eqi-potent from one strain to another, while **4** is specifically more active on *H. coffeaeformis*. Aplyzanzine E (**3**) and purealidin A (**5)** are stronger bacterial adhesion inhibitors, with an activity comparable to ianthelline on *V. aeturianus*. Only compounds **3** and **6** are shown to be bacterial growth inhibitors, with a special impact on *V. aestuarianus*, and an efficacy comparable to synoxazolidinone A for compound **3**. Compound **6** has the same range of activity as synoxazolidinone C and ianthelline with respect to this bacterium.

## 3. Materials and Methods 

### 3.1. General Experimental Procedures

Optical rotations were measured using an MCP-300 polarimeter (Anton Paar, Les Ulis, France). 1D and 2D NMR spectra were recorded on Avance 500 and 600 MHz spectrometers (Bruker, Wissembourg, France). The chemical shifts are relative to the residual signal solvent (CD_3_OD: δ_H_ 3.31; δ_C_ 49.20). High-resolution mass spectra were obtained on an LCT Premier XE spectrometer (Waters, Guyancourt, France) in electrospray ionization mode by direct infusion of the purified compounds. Chromatography on silica gel columns, was carried out on silica (Macherey-Nagel 60, 40–63 µm, Hoerdt, France), using a gradient of cyclohexane, ethyl acetate, methylene chloride, and methanol (Sigma-Aldrich, Saint-Quentin Fallavier, France). High performance liquid chromatography was performed on a 1260 Infinity system (Agilent, Les Ulis, France) equipped with a photodiode array detector (G1315C), an evaporative light scattering detector (G4260B), using an analytical reversed phase column Uptisphere C_18_ (4.6 mm × 250 mm, 5 µm, Interchim, Montluçon, France), and a semi-preparative Uptishere C_18_ (7.8 × 250 mm, 5 µm). 

### 3.2. Animal Material

The sponge referenced under the number P281-TRAR04 was collected in the Tuamotu archipelago in French Polynesia during the sampling cruise Tuam’2011 [73] aboard the R/V Alis, off the coast of Raroia (2011/05/13, 16°00.551’S; 142°27.243’W), using SCUBA between 45 and 55 m deep. It was identified as *Pseudoceratina* sp. (OTU QM2081) and a reference specimen is deposited at the Queensland Museum (Brisbane, Australia) under the accession number QM G333336.

Samples (230 g) were deep frozen immediately aboard and kept at −20°C until processing to obtain deliver 86 g of freeze-dried powder. 

### 3.3. Isolation of Bioactive Compounds

30 g of freeze-dried sponge were extracted by maceration at room temperature with 150 mL of EtOH/H_2_O (80:20) for 90 minutes, and then twice with 150 mL of EtOH (96%) for 45 minutes. The extracts were combined and the ethanol was evaporated under reduced pressure. A liquid–liquid partition between CH_2_Cl_2_ and H_2_O was done on the resulting residue. After drying of the organic layer upon MgSO_4_, solvents were evaporated under reduced pressure to offer the extract C (1.49 g, 4.9%). A precipitate at the interface between the organic and the aqueous layer was saved and dried (extract P, 1.28 g, 4.21%). The aqueous layer was partitioned against n-BuOH, to deliver the organic extract B (3.62 g, 12%) after drying and evaporation of the solvents.

The precipitate P (150 mg) was fractionated on normal-phase silica SPE (Strata, 1 g, 55 µm, 70 Å, Phenomenex, Le Pecq, France,), using the gradient from 1:0 to 0:1 mixture of cyclohexane/AcOEt then 1:0 to 0:1 mixture of CH_2_Cl_2_/MeOH to deliver finally six fractions. The second fraction (34 mg; 10% MeOH), the third (21 mg; 10%–12% MeOH) and the fourth (24 mg: 14%–20% MeOH) were submitted to further purification by semi-preparative reversed-phase HPLC (H_2_O/CH_3_CN/0.1%TFA). Aplyzanzine C (**1**) was isolated from each fraction but aplyzanzine E (**3**) only from the second fraction and **6** from the third one.

Extract C (500 mg) was subjected to normal-phase silica-gel chromatography, using the gradient from 1:0 to 0:1 mixture of cyclohexane/AcOEt then 1:0 to 0:1 mixture of CH_2_Cl_2_/MeOH successively to produce 13 fractions. After QSi evaluation on *V. harveyi*, only the third fraction (83 mg; 50% AcOEt) and the fourth (51 mg; 80% AcOEt) showed a biological activity. Respectively, 20 and 10 mg of these two fractions were further purified by semi-preparative reversed-phase HPLC (H_2_O/CH_3_CN/0.1%TFA). The aplyzanzine D (**2**) was the main product isolated, but aplyzanzine F (**4**) was obtained as a minor compound only from the 50% AcOEt fraction.

The extract B (500 mg) was subjected to gel-permeation chromatography on Sephadex LH20, using MeOH as solvent to finally deliver nine fractions. A portion (25 mg) of the sixth fraction (135 mg) was further purified by semi-preparative reversed-phase HPLC (H_2_O/CH_3_CN/0.1%TFA) to offer aplyzanzine C (**1**), aplyzanzine E (**3**), purealidin A (**5**) and (**6**).

*Aplyzanzine C* (**1**): colourless varnish (25.7 mg); ${\left[\alpha \right]}_{D}^{25}$ +26° (*c* 1.0, MeOH); UV (MeOH) λ_max_ (log ε) 207 (4.79) nm; ^1^H and ^13^C NMR data, Table 1; HRESIMS *m/z* = 729.9170 [M + H]^+^ (calcd. for C_24_H_32_N_3_O_3_^79^Br_2_^81^Br_2,_ 729.9136)

*Aplyzanzine D* (**2**): colourless varnish (16.7 mg); ${\left[\alpha \right]}_{D}^{25}$ +27.4° (*c* 1.0, MeOH); UV (MeOH) λ_max_ (log ε) 206 (4.77) nm; ^1^H and ^13^C NMR data, Table 1; HRESIMS *m/z* = 672.8610 [M+H]^+^ (calcd. for C_21_H_25_N_2_O_3_^79^Br_2_^81^Br_2,_ 672.8558).

*Aplyzanzine E* (**3**): colourless compound (1.4 mg): UV (MeOH) λ_max_ (log ε) 207 (4.48), 284 (3.79) nm; ^1^H and ^13^C NMR data, Table 2; HRESIMS *m/z* = 684.8616 [M + H]^+^ (calcd. for C_22_H_25_N_2_O_3_^79^Br_2_^81^Br_2,_ 684.8558).

*Aplyzanzine F* (**4**): colourless varnish (1.0 mg): UV (MeOH) λ_max_ (log ε) 208 (4.83), 285 (4.15) nm; ^1^H and ^13^C NMR data, Table 2; HRESIMS *m/z* = 627.8020 [M + H]^+^ (calcd. for C_19_H_18_NO_3_^79^Br_2_^81^Br_2,_ 627.7979).

*Purealidin A* (**5**): colourless compound (1.5 mg): ^1^H NMR data, Supplementary Materials; HRESIMS *m/z* = 519.0208 [M + H]^+^ (calcd. for C_17_H_23_N_6_O_3_^79^Br^81^Br, 519.0178).

**6**: colourless compound (4.0 mg): ^1^H NMR data, Supplementary Materials; HRESIMS *m/z* = 444.9745 [M + H]^+^ (calcd. for C_15_H_17_N_4_O_2_^79^Br^81^Br, 444.9698).

### 3.4. QSi Assay

The extracts and compounds were screened on agar plate using the wild *V. harveyi* strain CIP 104774. The 96-well microplates in liquid broth media was used for the bioguided isolation using several *Vibrio harveyi* strains [52]: *V. harveyi* BB120 wild-type and its derived double mutants (JAF 375, JMH 597, and JMH 612) obtained from Bassler’s laboratory [59,60,61], triplicates being performed for each sample tested.

### 3.5. Antifouling Assay

#### 3.5.1. Assays Against Marine Bacteria

All compounds were tested against three marine bacterial strains commonly found in biofilms, *Vibrio proteolyticus* (ATCC 15338), *Vibrio aesturianus* (ATCC 35048), and *Polaribacter irgensii* (ATCC 700398) [74]. Bacterial adhesion and growth rates were determined according to the method of Trepos et al. [75]. All assays were replicated 6 times.

Prior to experiments, bacteria were pre-grown in sterile Marine Bacterial Medium (MBM) with 0.5% peptone (neutralized bacteriological peptone, Oxoid Ltd, Basingstoke, UK) in sterile filtered (Whatman 1001-270, pore size 11-μm, GE Healthcare, Velizy-Villacoublay, France) natural seawater, at 20 °C.

Bacterial suspensions (100 µL aliquots, 2 × 10^8^ colony forming units/mL) were aseptically added to the microplate wells containing compound (0.01–10 µg/mL), and the plates were incubated for 48 h at 21 °C prior to assessment of bioactivity. Media only (Marine Broth 2216, Difco, BD Life Sciences, Le Pont de Claix, France) was used as a blank, and a commercial antifoulant (Seanine 211) was used as a positive control. 

Bacterial growth was monitored spectroscopically at 630 nm. Results are expressed as the minimal inhibitory concentration (MIC) for bacterial growth, which is defined as the lowest concentration which results in a decrease in OD, compared to the blank. The bacterial solutions were emptied from the microplates, and the bacterial adhesion assay was performed using aqueous crystal violet staining method on the microplates. The MIC for bacterial adhesion was defined as the lowest concentration of compound that, after 48-h incubation, produced a decrease of the OD at 595 nm compared to the blank.

#### 3.5.2. Assays Against Marine Microalgae

All compounds were tested for their capability to inhibit the adhesion and/or growth of 3 microalgal species known to be involved in surface colonization: *Cylindrotheca closterium* (AC 170), *Halamphora coffeaeformis* (AC 713), and *Porphyridium purpureum* (AC 122) [75]. All the strains were obtained from the Algobank culture collection (France). Prior to experiments, microalgae were grown in sterile F/2 medium (Guillard and Ryther, 1962) for 7 days at room temperature, exposed to natural daylight irradiance. Microalgal adhesion and growth rates were determined according to the method of Trepos et al. [75]. All assays were replicated 6 times.

In order to evaluate the algal growth and adhesion inhibition, compounds were diluted in methanol and transferred to black 96-multiwell plates. After evaporation of the carrier solvent, F/2 medium was added to reach the same final concentrations tested for the antibacterial activity. The same controls than for the antibacterial test were carried out. Microalgal stock solutions were prepared using the chlorophyll analysis methodology described by Chambers et al. (2011) [76]. The pre-treated microplate wells were treated with 100 μL of the algal stock solutions (0.1 mg chlorophyll a/L). The plates were then incubated for one week at room temperature, exposed to the daylight irradiance. Growth was determined by analysis of liberated chlorophyll a after centrifugation and methanol addition. Chlorophyll a was quantified fluorometrically (Tecan Infinite M200, excitation 485 nm, emission 645 nm). Results are expressed as the minimal inhibitory concentration (MIC) for microalgal growth which is defined as the lowest concentration which results in a decrease in OD, compared to the blank. 

Microalgal adhesion was determined in an analogous manner where the non-attached algal cells were removed prior to methanol addition (100 μL), releasing chlorophyll a from the remaining algal biofilms. Results were expressed as MIC values.

## 4. Conclusions

Four new bromotyrosine metabolites, the aplyzanzines C–F (**1**–**4**), along with the known 2-aminoimidazolic compounds, purealidin A (**5**) and **6,** were isolated and identified from the French Polynesian sponge *Pseudoceratina* sp. 2081. Biological evaluations were carried out to measure the capacity to inhibit *V. harveyi*’s QS and antifouling activities against 3 strains of microalgae and 3 strains of marine bacteria. Aplyzanzine E (**3**), purealidin A (**5**) and (**6**) displayed QSi activity against *V. harveyi*, and also against fouling bacteria. To the best of our knowledge, QSi activity of this type of bromotyrosine derivatives is described here for the first time. Aplyzanzine E (**3**) and purealidin A (**5**) reduced the adhesion of bacteria with a MIC smaller than 0.2 µM. Aplyzanzine E (**3**), purealidin A (**6**) inhibited the growth of *V. aesturianus*, with MIC, respectively, of 0.01 and 0.22 µM. Aplyzanzine C (**1**) was found to be the most active of these compounds against microalgae: it inhibits adhesion and growth of the three microalgae strains tested with a MIC of 0.14 µM. Aplyzanzine F (**4**) and purealidin A (**5**) are specific to *H. coffeaeformis*, inhibiting adhesion and growth, respectively, with a MIC of 0.02 µM. 

Defence synergies combining physical, mechanical or chemical parade are part of the strategies of living systems to survive in nature. This also explains why some marine organisms synthesize an arsenal of chemicals that will have in the same time targeted- and broad-spectrum activities. The biosynthesis of some of these compounds is constitutive, while for others, the synthesis will be induced by the season or by the presence of colonizing organisms. In this study, we found that compounds **1** and **3** have very complementary activities that allow the sponge *Pseudoceratina* sp. 2081 to fight microfouling in a holistic way. 

## Figures and Tables

**Table 1:** ^1^H NMR (500 MHz) and ^13^C NMR (125 MHz) data for aplyzanzines C (**1**) and D (**2**) in CD_3_OD and aplyzanzine A data (in CDCl_3_ + CD_3_OD (10:1)) from the literature.

Position	Aplyzanzine C (1)		Aplyzanzine D (2)		Aplyzanzine A	
No.	δ_C_, Type	δ_H,_ Mult	δ_C_, Type	δ_H,_ Mult	δ_C_, Type	δ_H,_ Mult (*J* in Hz)
1	168.3, C	-	167.2, C	-	170.8, C	-
2	66.3, CH	4.65, m	65.9, CH	4.41, m	69.8, CH	3.14, dd (4.5, 8.8)
3	35.1, CH_2_	2.97–3.47, m	30.4, CH_2_	2.93–3.30, m	31.6, CH_2_	2.71, dd (4.5, 13.8) 2.94, dd (8.8, 13.5)
4	134.0, C	-	132.0, C	-	137.6, C	-
5, 9	135.6, CH	7.55, s	133.6, CH	7.36, s	133.2, CH	7.31, s
6, 8	119.4, C	-	110.5, C	-	117.6, C	-
7	155.7, C	-	149.2, C	-	152.3, C	-
10, 11	42.7, CH_3_	2.91, s	42.1, CH_3_	2.71, br s	41.51, CH_3_	2.26, s
12	61.4, CH_3_	3.84, s			61.4, CH_3_	3.74, s
1’	50.5, CH_2_	3.47, m	50.4, CH_2_	3.54, m	39.8, CH_2_	3.28, dt (2.8, 7.2)
2’	32.8, CH_2_	2.57, m	32.4, CH_2_	2.53, m	34.2, CH_2_	2.54, m 2.27, m
3’	139.5, C	-	133.9, C	-	137.9, C	-
4’, 8’	134.5, CH	7.51, s	135.1, CH	7.54, s	132.8, CH	7.23, s
5’, 7’	119.1, C	-	117.3, C	-	117.7, C	-
6’	152.7, C	-	153.4, C	-	150.9, C	-
9’	35.8, CH_3_	2.65, s	35.4, CH_3_	2.64, s		
10’			61.0, CH_3_	3.83, s		
1”	71.8, CH_2_	4.11, m			69.7, CH_2_	3.96, t (5.5)
2”	29.2, CH_2_	2.21, m			25.4, CH_2_	2.18, m
3”	39.0, CH_2_	3.27, m			55.4, CH_2_	3.16, m
4”, 5”					42.92, CH_3_	2.67, s

**Table 2:** ^1^H NMR (500 MHz) and ^13^C NMR (125 MHz) data for aplyzanzines E (**3**) and F (**4**) in CD_3_OD.

Position	Aplyzanzine E (3)		Aplyzanzine F (4)	
No.	δ_C_, Type	δ_H,_ Mult (*J* in Hz)	δ_C_, Type ^1^	δ_H,_ Mult (*J* in Hz) ^1^
1	168.9, C	-	**169.2**/168.4, C	-
2	120.7, CH	6.60, d (15)	**120.7**/121.2, CH	**6.64, d (16)**/7.09, d (16)
3	138.4, CH	6.96, d (15)	**138.8**/140.4, CH	**6.97, d (16)**/7.38, d (16)
4	133.3, C	-	135.6, C	-
5, 9	132.9, CH	7.69, s	**133.2**/133.4, CH	**7.68, s**/7.90, s
6, 8	119.9, C	-	119.7, C	-
7	156.5, C	-	156.2, C	-
10	61.1, CH_3_	3.88, s	61.3, CH_3_	3.87, s
1’	52.2, CH_2_	3.85, m	**52.6**/51.5, CH_2_	**3.78, t (6)**/3.63, t (6)
2’	34.2, CH_2_	2.86, m	**34.0**/31.9, CH_2_	**2.79, m**/2.78, m
3’	135.2, C	-	133.1, C	-
4’, 8’	134.9, CH	7.40, s	**134.3**/133.8, CH	**7.27, s**/7.39, s
5’, 7’	119.2, C	-	112.5, C	-
6’	152.7, C	-	151.9, C	-
9’	34.2, CH_3_	3.09, s	**34.7**/36.9, CH_3_	**3.05, s**/3.16, s
1”	71.5, CH_2_	4.10, m		
2”	29.1, CH_2_	2.19, m		
3”	38.6, CH_2_	3.30, m		

^1^ Presence of two rotamers in a 70/30 ratio. In bold, the data of the predominant form.

**Table 3:** Luminescence curves of wild strain BB120 and its three derived double mutants (JAF 375, JMH 597, and JMH 612) in the presence of purified compounds tested at two concentrations or not (control).

^____^: Control without Product; - - -: 50 µg/mL; ^……^: 5 µg/mL				
No.	BB120	JAF 375 (CAI-1 +)	JMH 597 (AI-2 +)	JMH 612 (HAI-1 +)
**1**	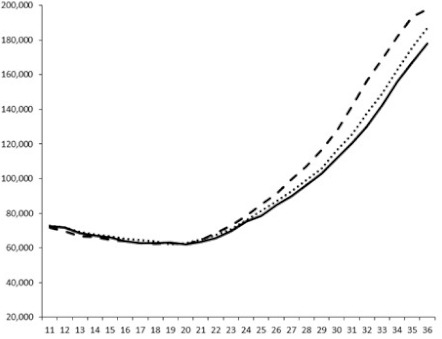	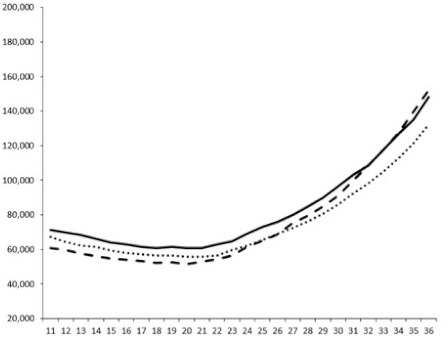	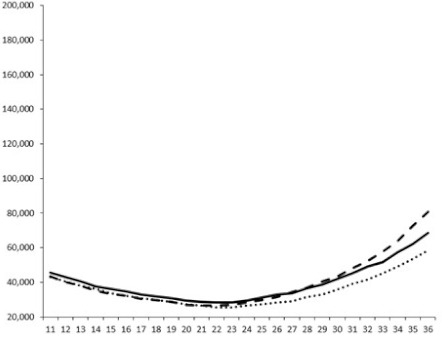	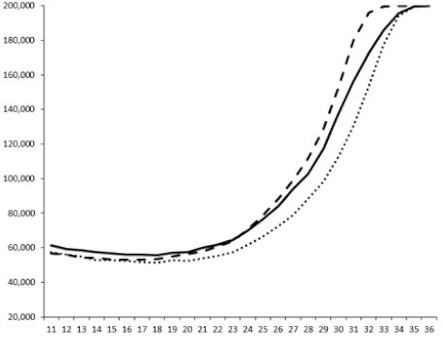
**2**	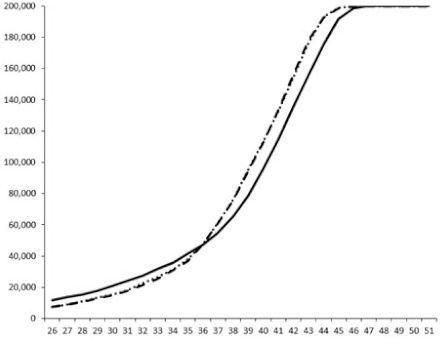	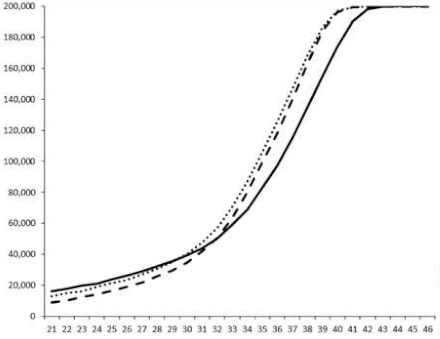	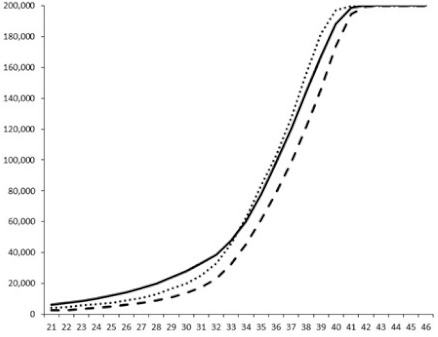	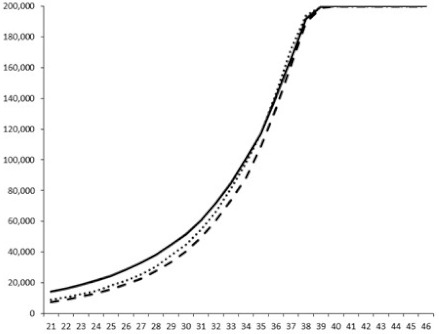
**3**	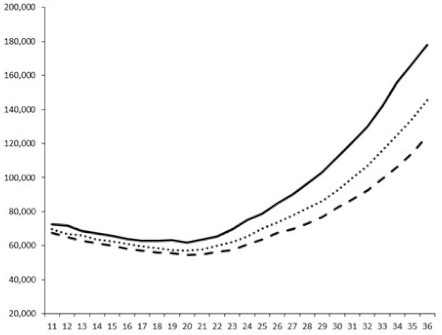	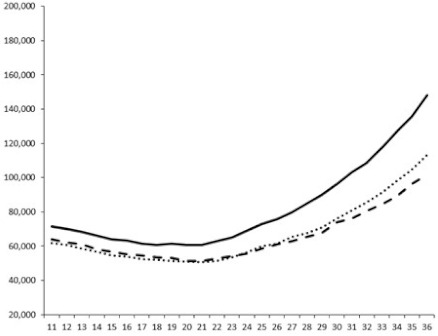	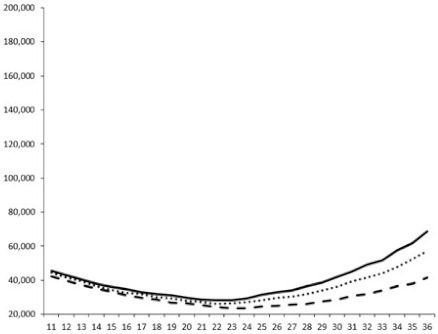	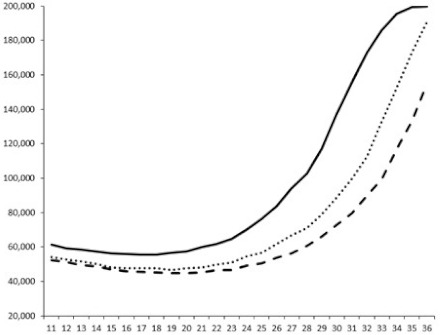
**4**	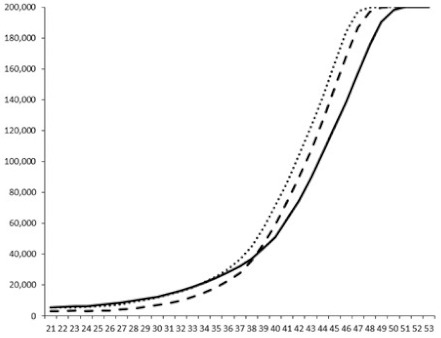	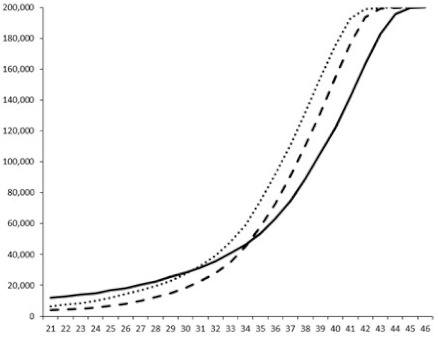	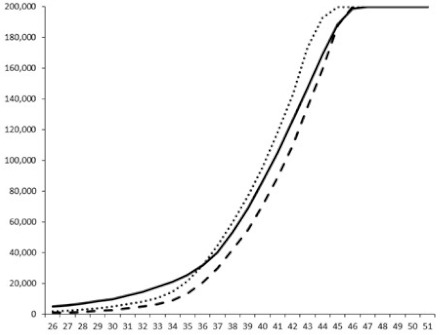	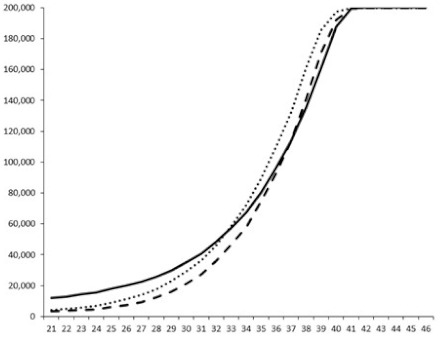
**5**	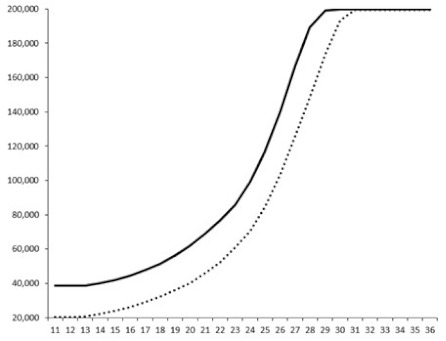	nt ^1^	nt	nt
**6**	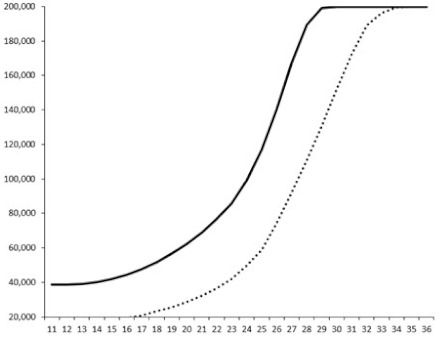	nt	nt	nt

^1^ nt = not tested; *Y*-axis: relative luminescence units (RLU); *X*-axis: *n* cycles of 10 min.

**Table 4:** Activities of the purified compounds **1**–**6** compared to Seanine 211, bromotyrosine derivatives taken from the literature against microorganisms involved in biofouling (MIC in µM).

	Microalgae			Marine Bacteria		
Mol.	*P. purpureum*	*C. closterium*	*H. coffeaeformis*	*V. proteolyticus*	*V. aestuarianus*	*P. irgensii*
*Adhesion inhibition*						
**1**	**0.14** ^1^	**0.14**	**0.14**	-	-	-
**2**	- ^2^	-	-	-	1.48	-
**3**	-	-	-	**0.15**	**0.15**	-
**4**	-	-	**0.02**	-	-	-
**5**	-	-	-	**0.02**	**0.19**	**0.19**
**6**	-	-	-	-	-	-
Seanine ^3^	**<0.** **03**	**<0.** **03**	**<0.** **03**	**0.** **03**	3.5	3.5
Syn. A ^4^	-	20	20	-	-	20
Syn. C ^5^	**0.2**	2	2	2	2	20
Pul. A ^6^	-	-	30	-	**0.03**	-
Pul. B ^7^	-	-	-	-	20	-
Ianthel. ^8^	-	-	-	-	**0.2**	-
*Growth inhibition*						
**1**	**0.14**	**0.14**	**0.14**	1.37	-	-
**2**	1.48	1.48	-	-	-	1
**3**	-	-	-	-	**0.01**	-
**4**	-	-	-	-	-	-
**5**	19	1.9	**0.02**	-	-	-
**6**	-	-	-	-	**0.22**	-
Seanine	**<0.** **03**	**<0.** **03**	**<0.** **03**	**0.** **03**	**<0.** **03**	3.5
Syn. A	20	20	20	**0.02**	**0.02**	-
Syn. C	**0.02**	**0.2**	2	**0.2**	**0.2**	2
Pul. A	**0.2**	-	-	-	-	**0.2**
Pul. B	-	-	-	-	-	-
Ianthel.	-	-	-	21	**0.2**	2.1

^1^ Numbers in bold represent the best biological activities; ^2^ compound inactive at ≥ 10 μg/mL concentrations; ^3^ Seanine 211; ^4^ synoxazolidinone A; ^5^ synoxazolidinone C; ^6^ pulmonarin A; ^7^ pulmonarin B; ^8^ ianthelline.

## Supplementary Materials

The following are available online at https://www.mdpi.com/1660-3397/18/5/272/s1. Spectroscopic data (HRMS, ^1^H NMR, ^13^C NMR, COSY, HSQC and HMBC) for compounds **1**–**6**.
