# Supplementary material for: Quorum Sensing Inhibitory and Antifouling Activities of New Bromotyrosine Metabolites from the Polynesian Sponge Pseudoceratina n. sp
Source: Mar Drugs. 2020 May 21;18(5):272. doi: 10.3390/md18050272 (PMC7281015; doi:10.3390/md18050272)

Supporting Information

Quorum Sensing Inhibitory and Antifouling Activities of New Bromotyrosine Metabolites from the Polynesian Sponge *Pseudoceratina* sp.

Florent Tintillier [1], Céline Moriou [2], Sylvain Petek [1,3,*], Marilyne Fauchon [3], Claire Hellio [3], Denis Saulnier [4], Merrick Ekins [5], John N. A. Hooper [5], Ali Al-Mourabit [2] and Cécile Debitus [1,3]

[1] IRD, Univ de la Polynésie française, Ifremer, ILM, EIO, F-98713 Papeete, French Polynesia; florent.tintillier@gmail.com (F.T.); cecile.debitus@ird.fr (C.D.)
[2] Université Paris-Saclay, CNRS, Institut de Chimie des Substances Naturelles, Gif-sur-Yvette, 91190, France; celine.moriou@cnrs.fr (C.M.); ali.almourabit@cnrs.fr (A.A.-M.)
[3] IRD, Univ Brest, CNRS, Ifremer, LEMAR, F-29280 Plouzane, France ; marilyne.fauchon@univ-brest.fr (M.F.) ; claire.hellio@univ-brest.fr (C.H.)
[4] Ifremer, IRD, ILM, Univ de la Polynésie française, EIO, F-98719 Taravao, French Polynesia ; denis.saulnier@ifremer.fr (D.S.)
[5] Queensland Museum, PO Box 3300, South Brisbane BC, Queensland 4101, Australia ; merrick.ekins@qm.qld.gov.au (M.E.) ; john.hooper@qm.qld.gov.au (J.H.)

* Correspondence: sylvain.petek@ird.fr (S.P.); Tel.: +33-298-498-651 (S.P.)

Table of Contents

Figure S1. ^{1}H NMR spectrum of Aplyzanzine C (**1**) in MeOD (500 MHz) 3
Figure S2. ^{13}C NMR spectrum of Aplyzanzine C (**1**) in MeOD (500 MHz) 4
Figure S3. ^{1}H-^{1}H COSY NMR spectrum of Aplyzanzine C (**1**) in MeOD (500 MHz) 5
Figure S4. HSQC NMR spectrum of Aplyzanzine C (**1**) in MeOD (500 MHz 6
Figure S5. HMBC NMR spectrum of Aplyzanzine C (**1**) in MeOD (500 MHz) 7
Figure S6. HR-ESI mass spectrum of Aplyzanzine C (**1**) 8
Figure S7. ^{1}H NMR spectrum of Aplyzanzine D (**2**) in MeOD (500 MHz) 9
Figure S8. ^{1}H-^{1}H COSY NMR spectrum of Aplyzanzine D (**2**) in MeOD (500 MHz) 10
Figure S9. HSQC NMR spectrum of Aplyzanzine D (**2**) in MeOD (500 MHz) 11
Figure S10. HMBC NMR spectrum of Aplyzanzine D (**2**) in MeOD (500 MHz) 12
Figure S11. HR-ESI mass spectrum of Aplyzanzine D (**2)** 13
Figure S12. ^{1}H NMR spectrum of Aplyzanzine E (**3**) in MeOD (600 MHz) 14
Figure S13. ^{1}H-^{1}H COSY NMR spectrum of Aplyzanzine E (**3**) in MeOD (600 MHz) 15
Figure S14. HSQC NMR spectrum of Aplyzanzine E (**3**) in MeOD (600 MHz) 16
Figure S15. HMBC NMR spectrum of Aplyzanzine E (**3**) in MeOD (600 MHz) 17
Figure S16. HR-ESI mass spectrum of Aplyzanzine E (**3**) 18
Figure S17. ^{1}H NMR spectrum of Aplyzanzine F (**4**) in MeOD (600 MHz) 19
Figure S18. ^{1}H-^{1}H COSY NMR spectrum of Aplyzanzine F (**4**) in MeOD (600 MHz) 20
Figure S19. HSQC NMR spectrum of Aplyzanzine F (**4**) in MeOD (600 MHz) 21
Figure S20. HMBC NMR spectrum of Aplyzanzine F (**4**) in MeOD (600 MHz) 22
Figure S21. HR-ESI mass spectrum of Aplyzanzine F (**4**) 23
Figure S22. ^{1}H NMR spectrum of **5** in MeOD (300 MHz) 24
Figure S23. HR-ESI mass spectrum of **5** 25
Table S1. Comparison of the spectroscopic data of the compound **5** with the data from the literature concerning the purealidin A[54] and the lipopurealin D[56]. 26
Figure S24: Lipopurealin D[56] and general key COSY for purealidin A in DMSO-*d6*.[54] 26
Figure S25. ^{1}H NMR spectrum of **6** in MeOD (500 MHz) 27
Figure S26. HR-ESI mass spectrum of **6** 28
Table S2. Comparison of the spectroscopic data of the compound **6** with the data from the literature.[55] 29
Figure S27: Structure of the compound 11 in Ciminiello et al.[55] 29

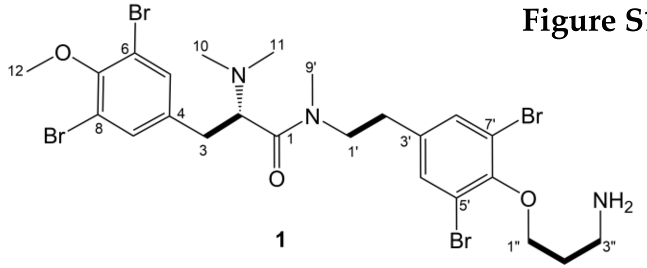

Figure S1. ^{1}H NMR spectrum of Aplyzanzine C (**1**) in MeOD (500 MHz)

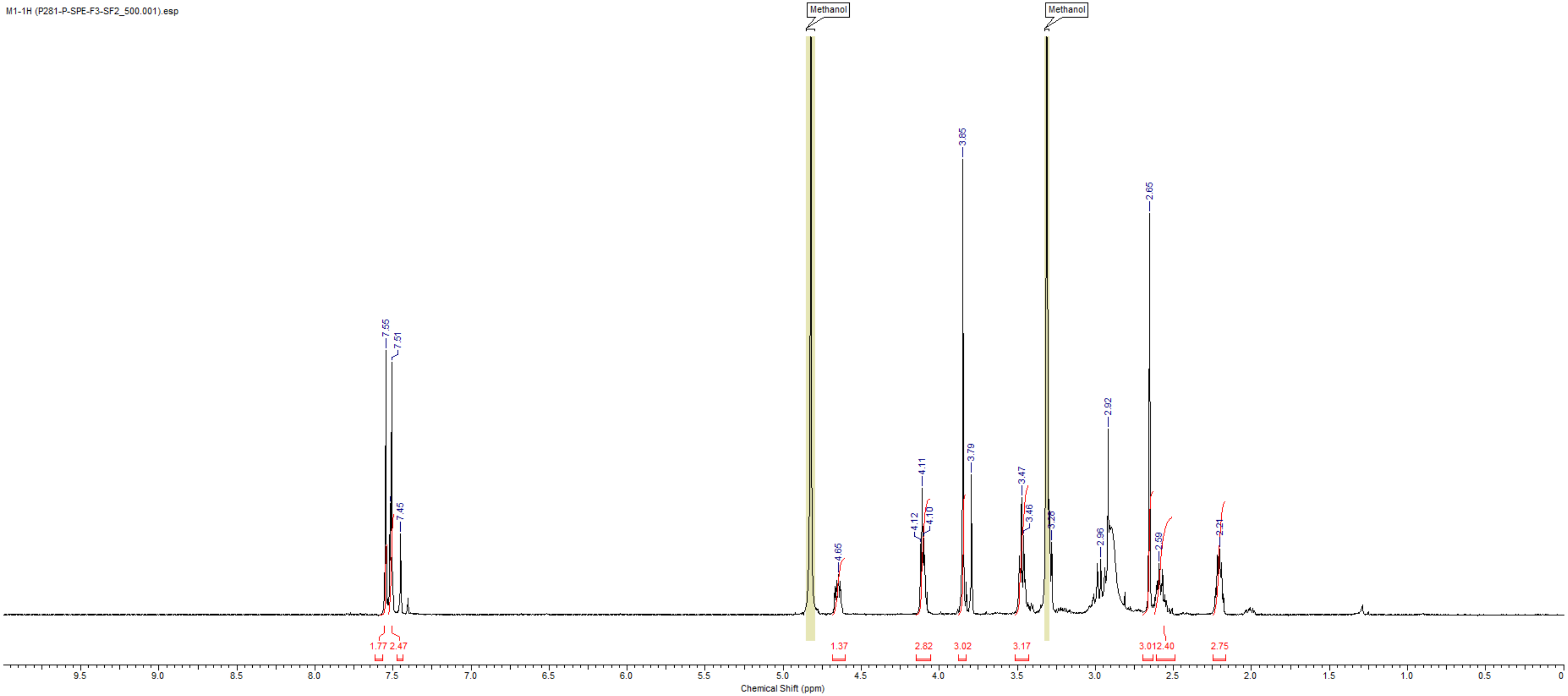

Figure S2. ^{13}C NMR spectrum of Aplyzanzine C (**1**) in MeOD (500 MHz)

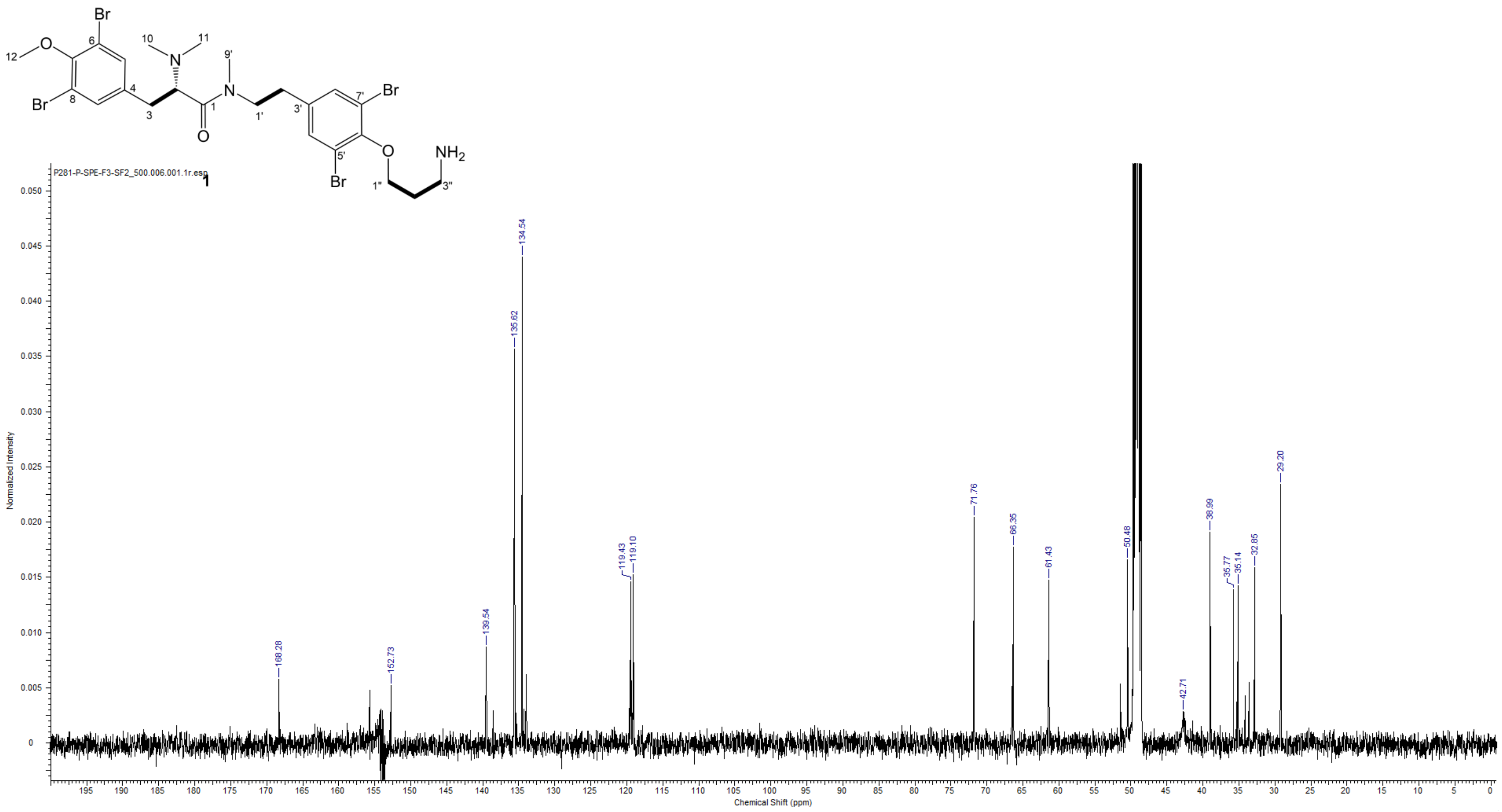

Figure S3. ^{1}H-^{1}H COSY NMR spectrum of Aplyzanzine C (**1**) in MeOD (500 MHz)

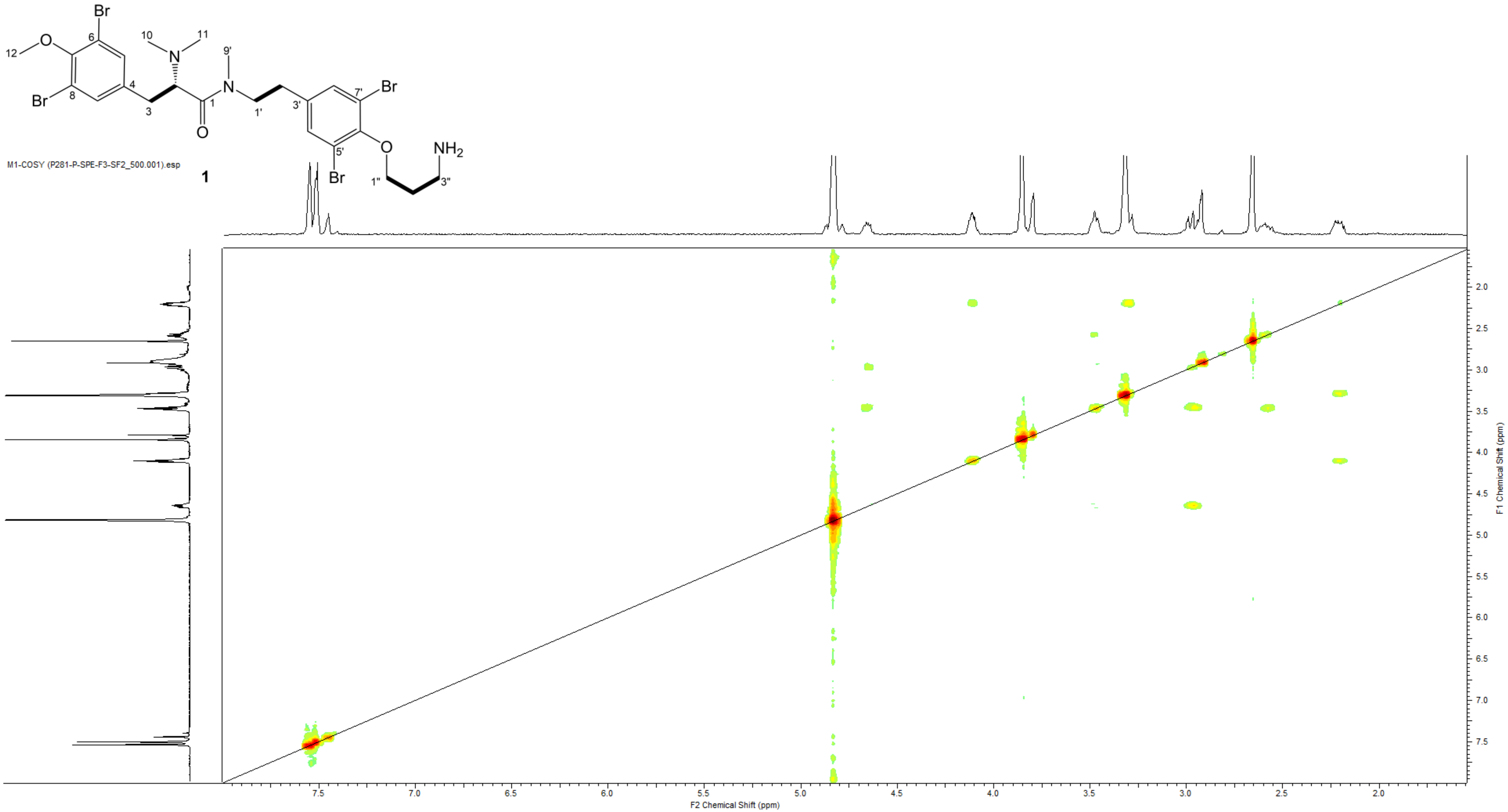

Figure S4. HSQC NMR spectrum of Aplyzanzine C (**1**) in MeOD (500 MHz)

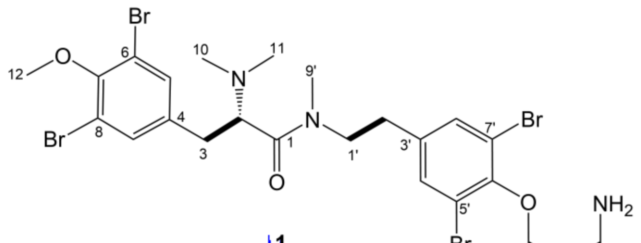

1

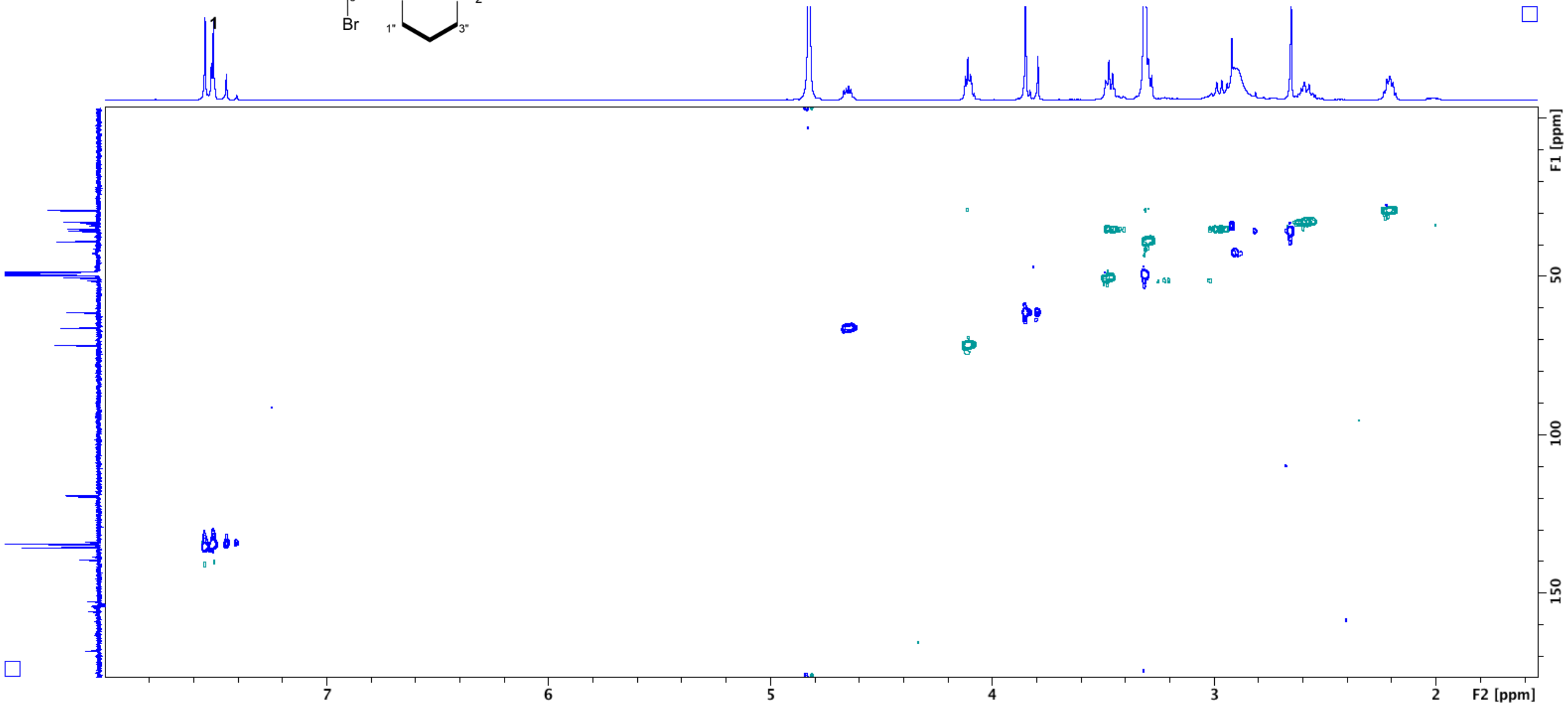

Figure S5. HMBC NMR spectrum of Aplyzanzine C (**1**) in MeOD (500 MHz)

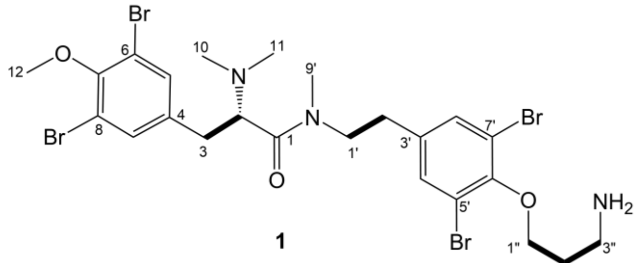

1

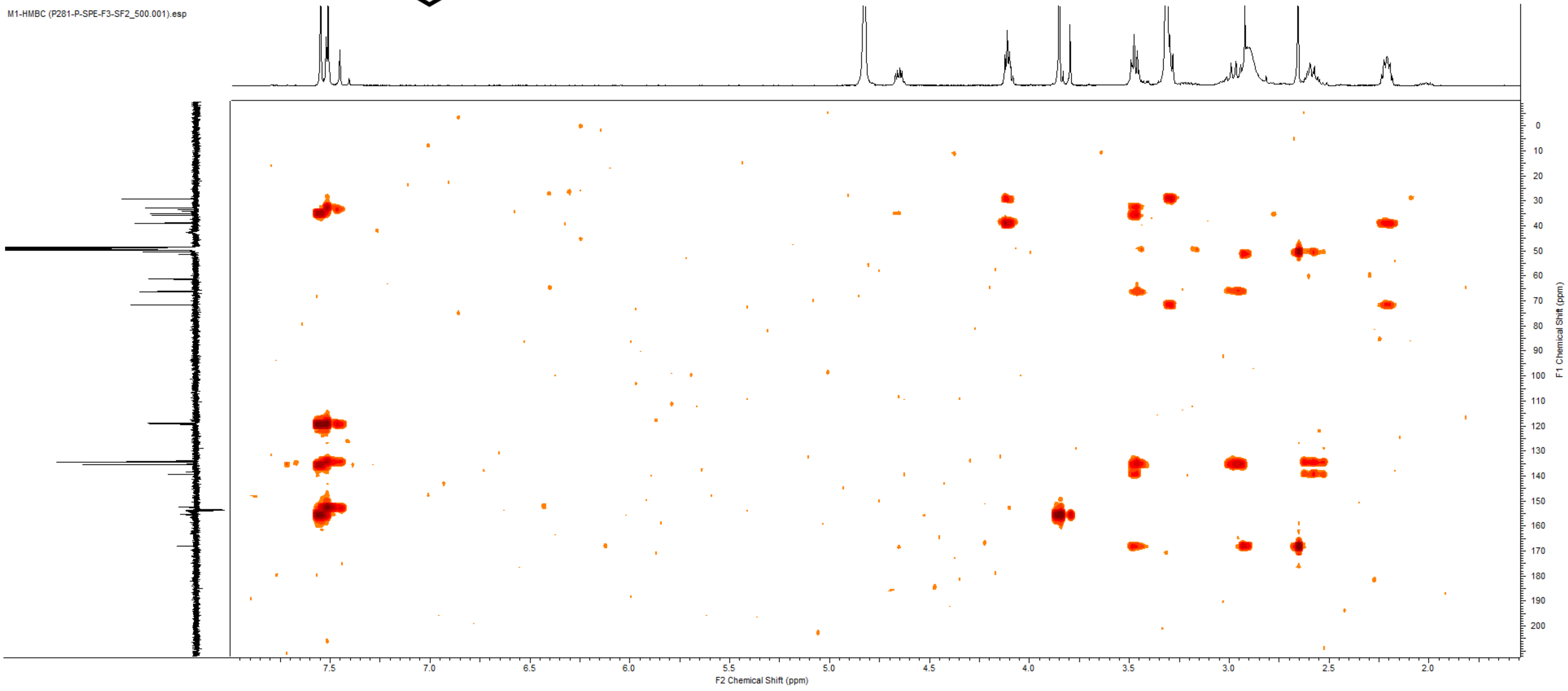

Figure S6. HR-ESI mass spectrum of Aplyzanzine C (**1**)

Elemental Composition Report

Page 1

Single Mass Analysis

Tolerance = 5.0 mDa / DBE: min = -1.5, max = 50.0
Element prediction: Off
Number of isotope peaks used for i-FIT = 3

Monoisotopic Mass, Even Electron Ions
2232 formula(e) evaluated with 7 results within limits (up to 50 best isotopic matches for each mass)
Elements Used:
C: 0-50 H: 0-100 N: 0-10 O: 0-10 79Br: 2-4 81Br: 2-4

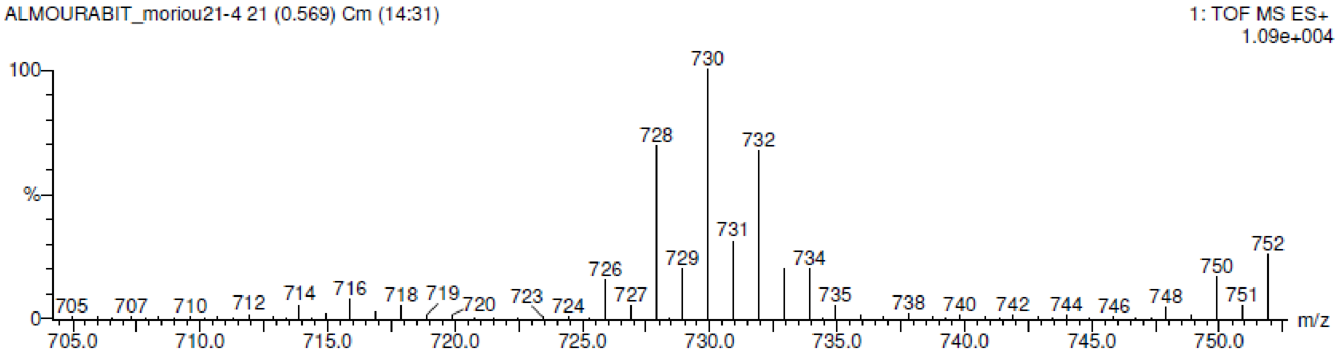

Minimum:				-1.5			
Maximum:		5.0	10.0	50.0			
Mass	Calc. Mass	mDa	PPM	DBE	i-FIT	i-FIT (Norm)	Formula
729.9140	729.9109	3.1	4.2	9.5	29.6	1.6	C20 H28 N9 O 79Br2 81Br2
	729.9168	-2.8	-3.8	0.5	29.7	1.7	C13 H32 N9 O6 79Br2 81Br2
	729.9096	4.4	6.0	4.5	29.7	1.8	C19 H32 N5 O5 79Br2 81Br2
	729.9122	1.8	2.5	-1.5	29.9	1.9	C16 H39 N7 79Br2 81Br3
	729.9136	0.4	0.5	8.5	30.0	2.0	C24 H32 N3 O3 79Br2 81Br2
	729.9115	2.5	3.4	1.5	30.4	2.4	C23 H41 N 79Br3 81Br2
	729.9176	-3.6	-4.9	12.5	30.5	2.5	C29 H32 N O 79Br2 81Br2

Figure S7. ^{1}H NMR spectrum of Aplyzanzine D (**2**) in MeOD (500 MHz)

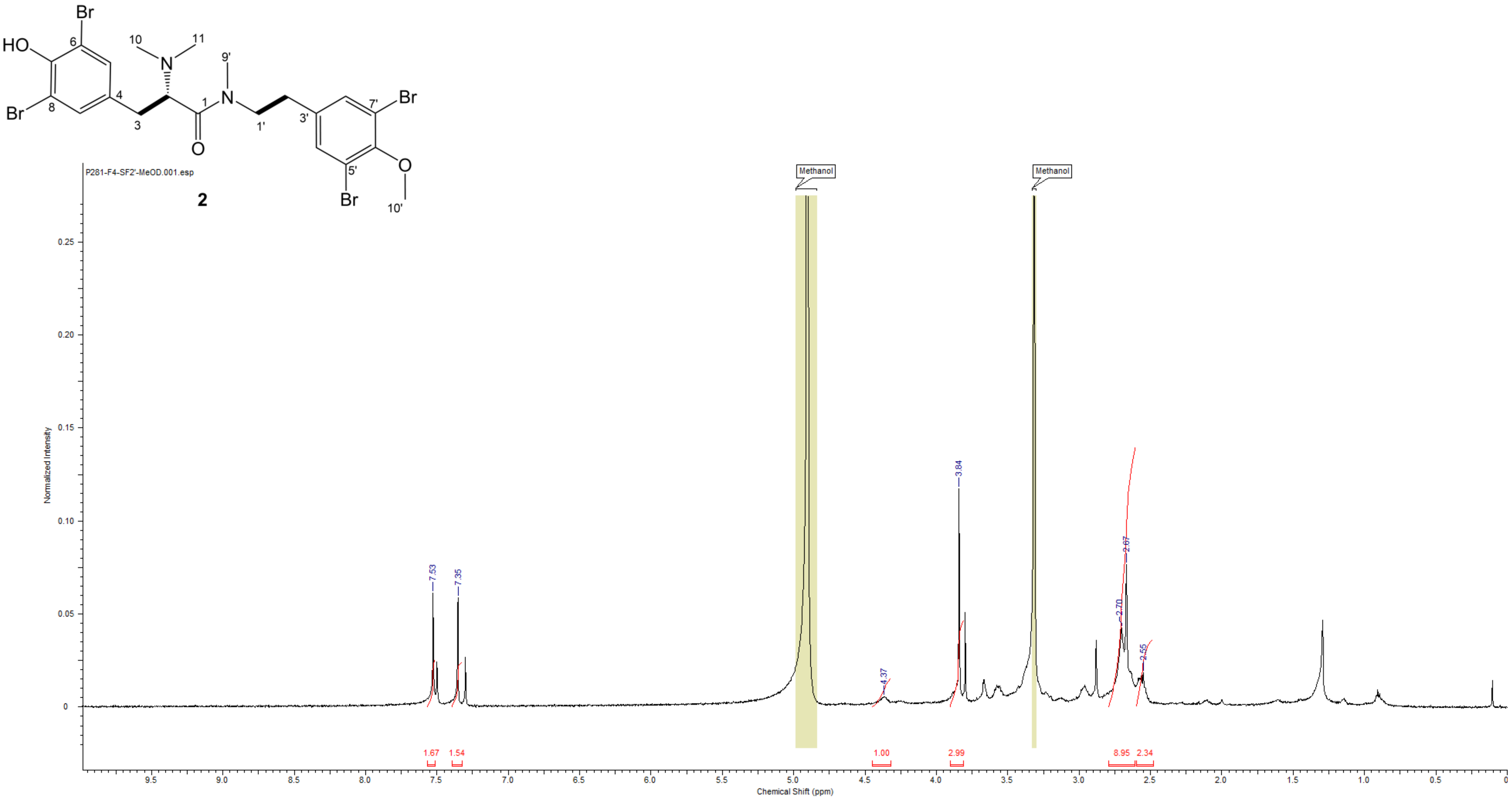

Figure S8. ^{1}H-^{1}H COSY NMR spectrum of Aplyzanzine D (**2**) in MeOD (500 MHz)

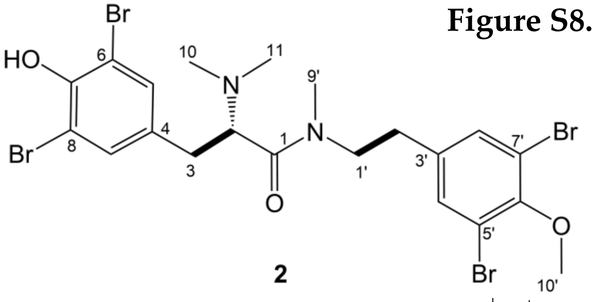

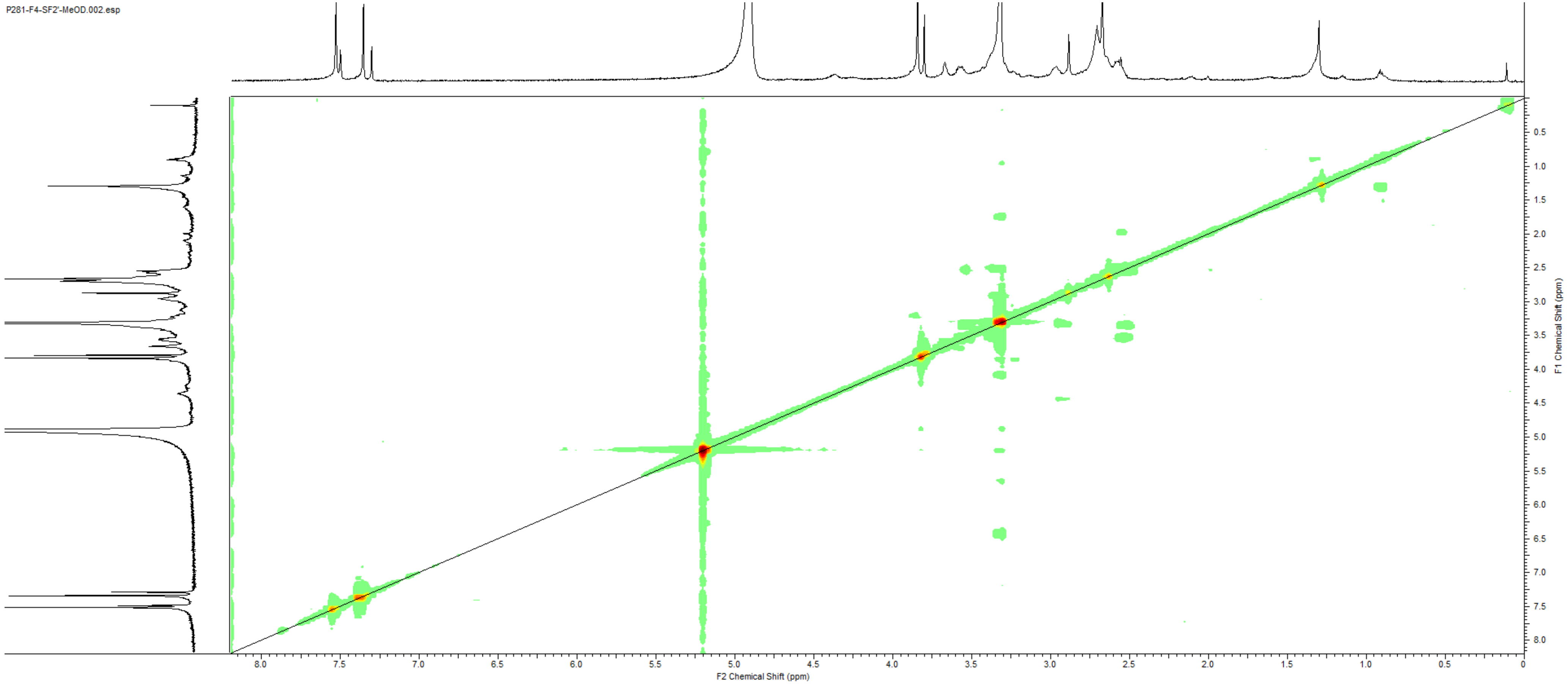

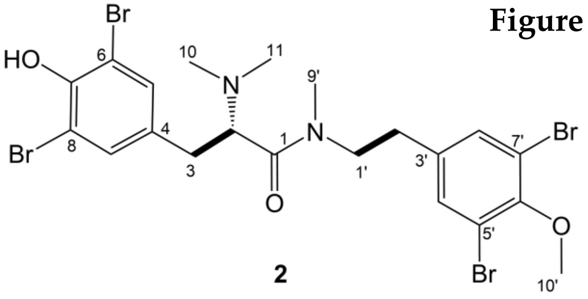

2

Figure S9. HSQC NMR spectrum of Aplyzanzine D (**2**) in MeOD (500 MHz)

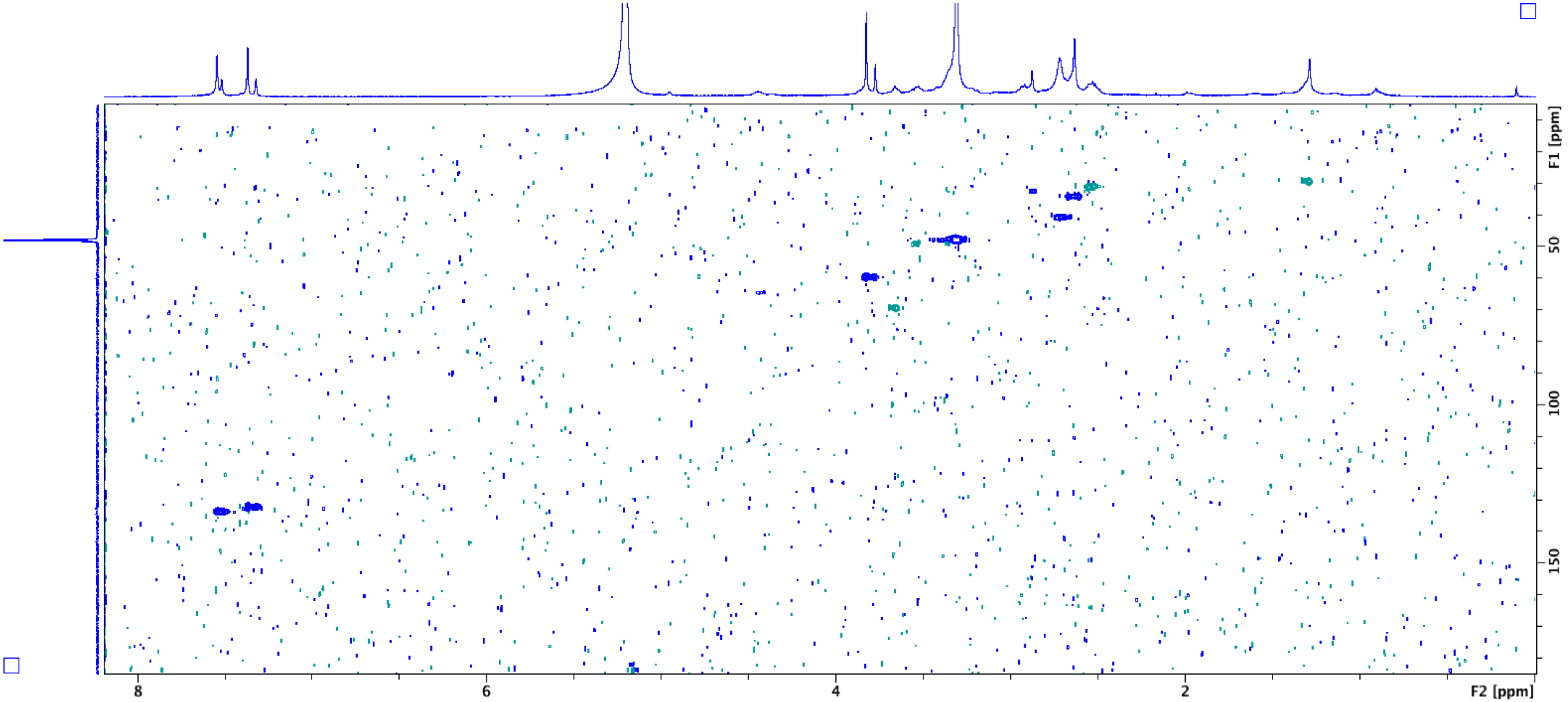

Figure S10. HMBC NMR spectrum of Aplyzanzine D (**2**) in MeOD (500 MHz)

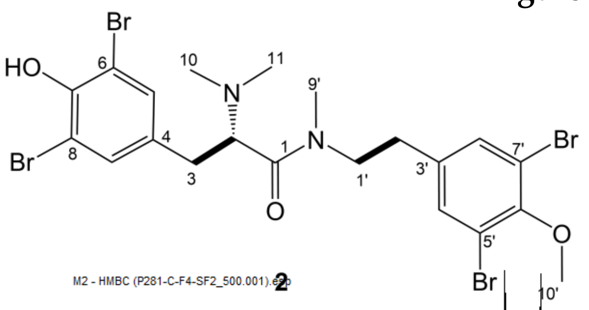

2

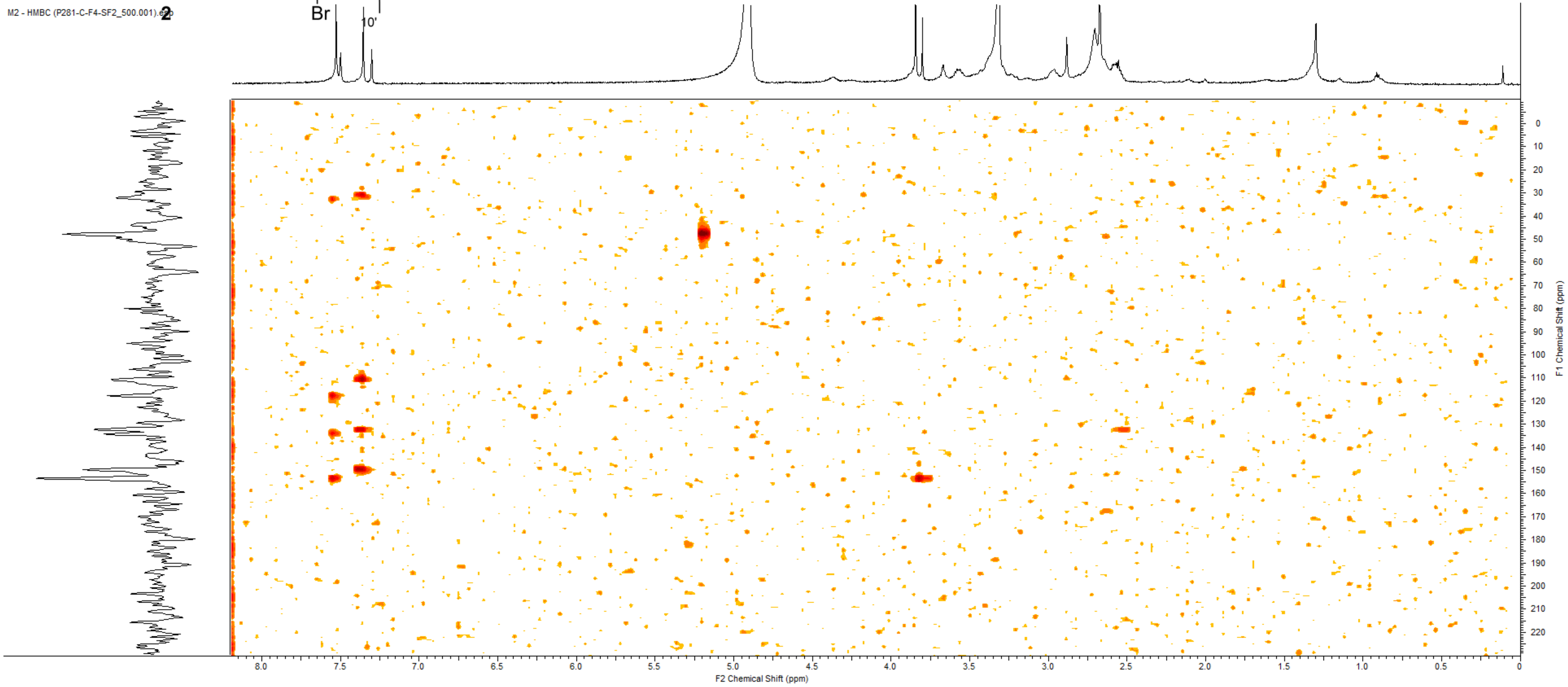

Figure S11. HR-ESI mass spectrum of Aplyzanzine D (**2**)

Elemental Composition Report

Single Mass Analysis

Tolerance = 50.0 mDa / DBE: min = -1.5, max = 50.0
Element prediction: Off
Number of isotope peaks used for i-FIT = 3

Monoisotopic Mass, Even Electron Ions
200 formula(e) evaluated with 29 results within limits (up to 50 closest results for each mass)
Elements Used:
C: 0-50 H: 0-100 N: 1-8 O: 2-8 79Br: 2-2 81Br: 2-2

ALMOURABIT_moriou32-1 21 (0.576) Cm (16:29) 1: TOF MS ES+ 5.80e+004

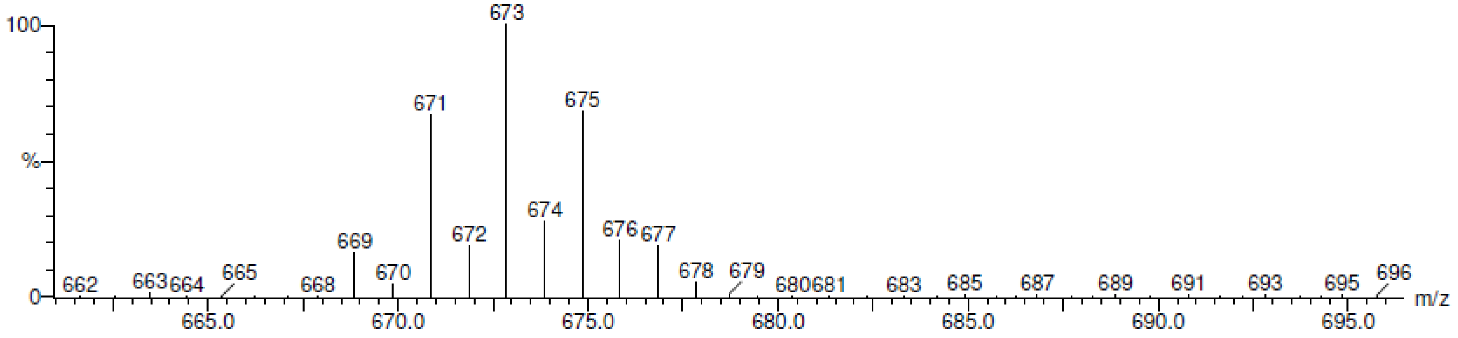

Minimum:				-1.5			
Maximum:		50.0	10.0	50.0			
Mass	Calc. Mass	mDa	PPM	DBE	i-FIT	i-FIT (Norm)	Formula
672.8610	672.8616	-0.6	-0.9	-0.5	38.5	3.3	C14 H29 N2 O8 79Br2 81Br2
	672.8630	-2.0	-3.0	4.5	38.5	3.3	C15 H25 N6 O4 79Br2 81Br2
	672.8590	2.0	3.0	0.5	38.5	3.4	C10 H25 N8 O6 79Br2 81Br2
	672.8558	5.2	7.7	8.5	38.5	3.4	C21 H25 N2 O3 79Br2 81Br2
	672.8670	-6.0	-8.9	8.5	38.5	3.4	C20 H25 N4 O2 79Br2 81Br2
	672.8517	9.3	13.8	4.5	38.5	3.4	C16 H25 N4 O5 79Br2 81Br2

Figure S12. ^{1}H NMR spectrum of Aplyzanzine E (**3**) in MeOD (600 MHz)

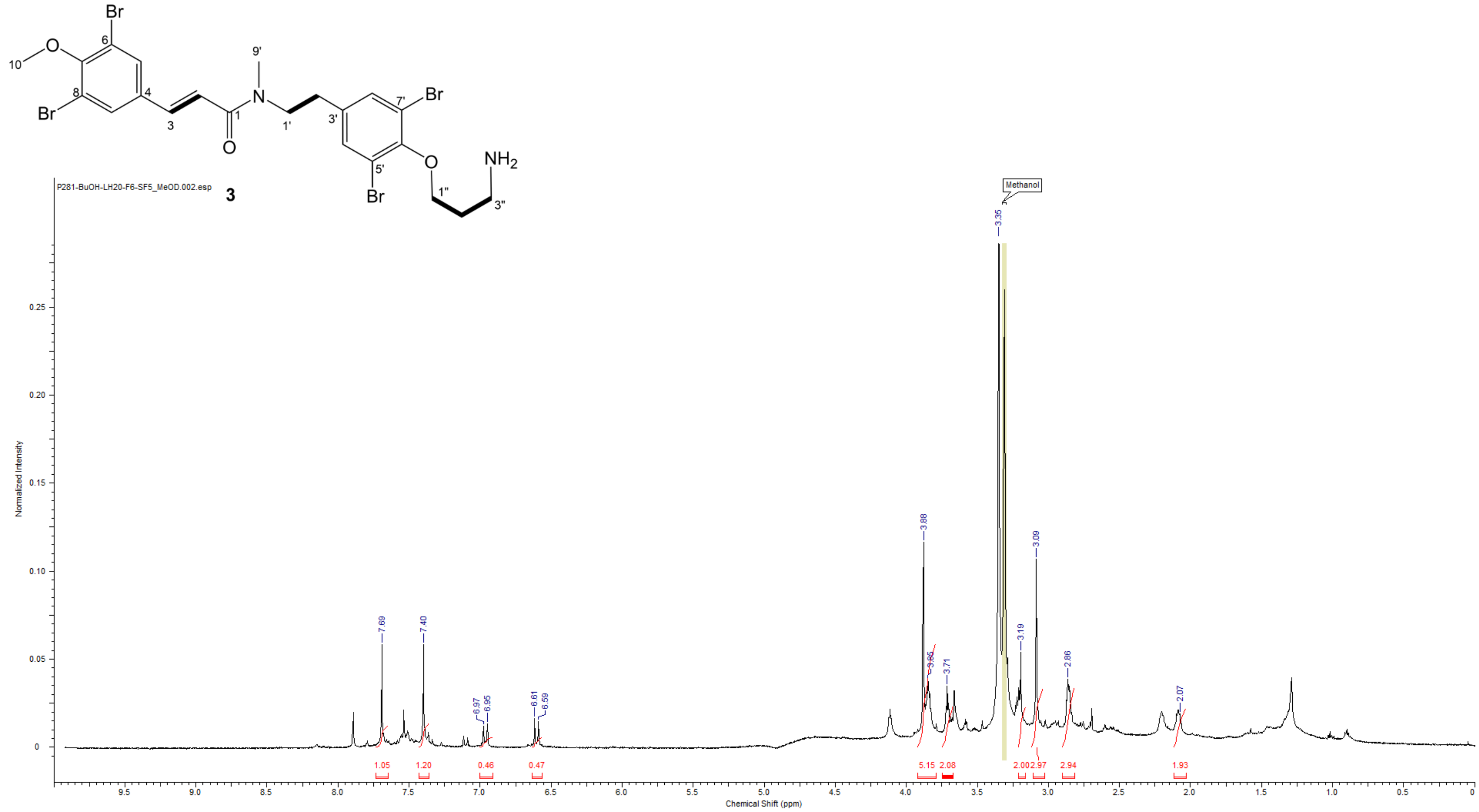

Figure S13. ^{1}H-^{1}H COSY NMR spectrum of Aplyzanzine E (**3**) in MeOD (600 MHz)

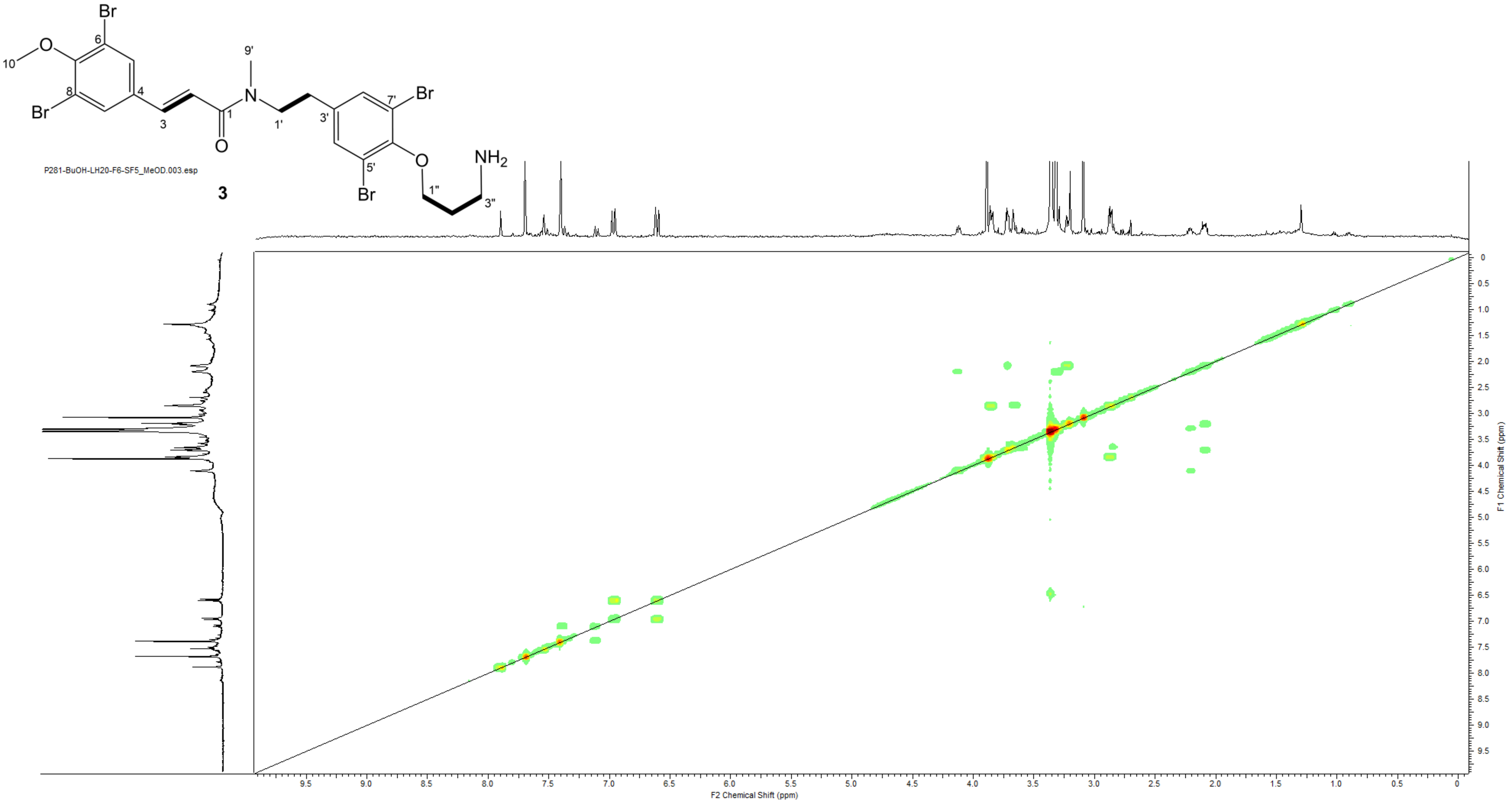

Figure S14. HSQC NMR spectrum of Aplyzanzine E (**3**) in MeOD (600 MHz)

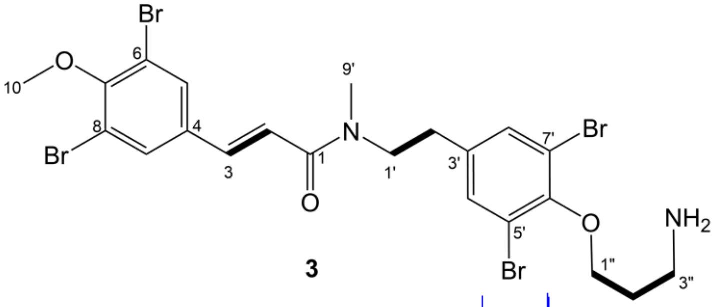

3

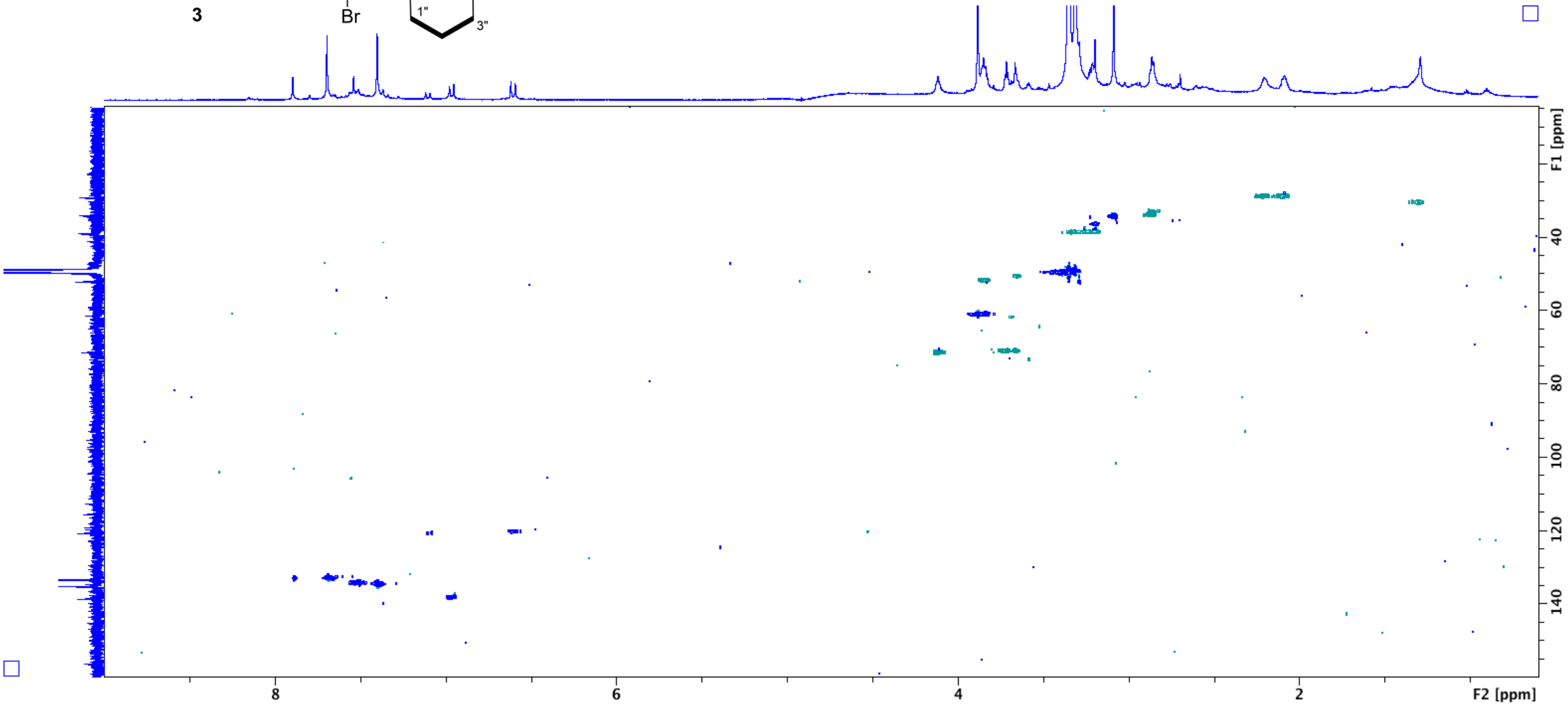

Figure S15. HMBC NMR spectrum of Aplyzanzine E (**3**) in MeOD (600 MHz)

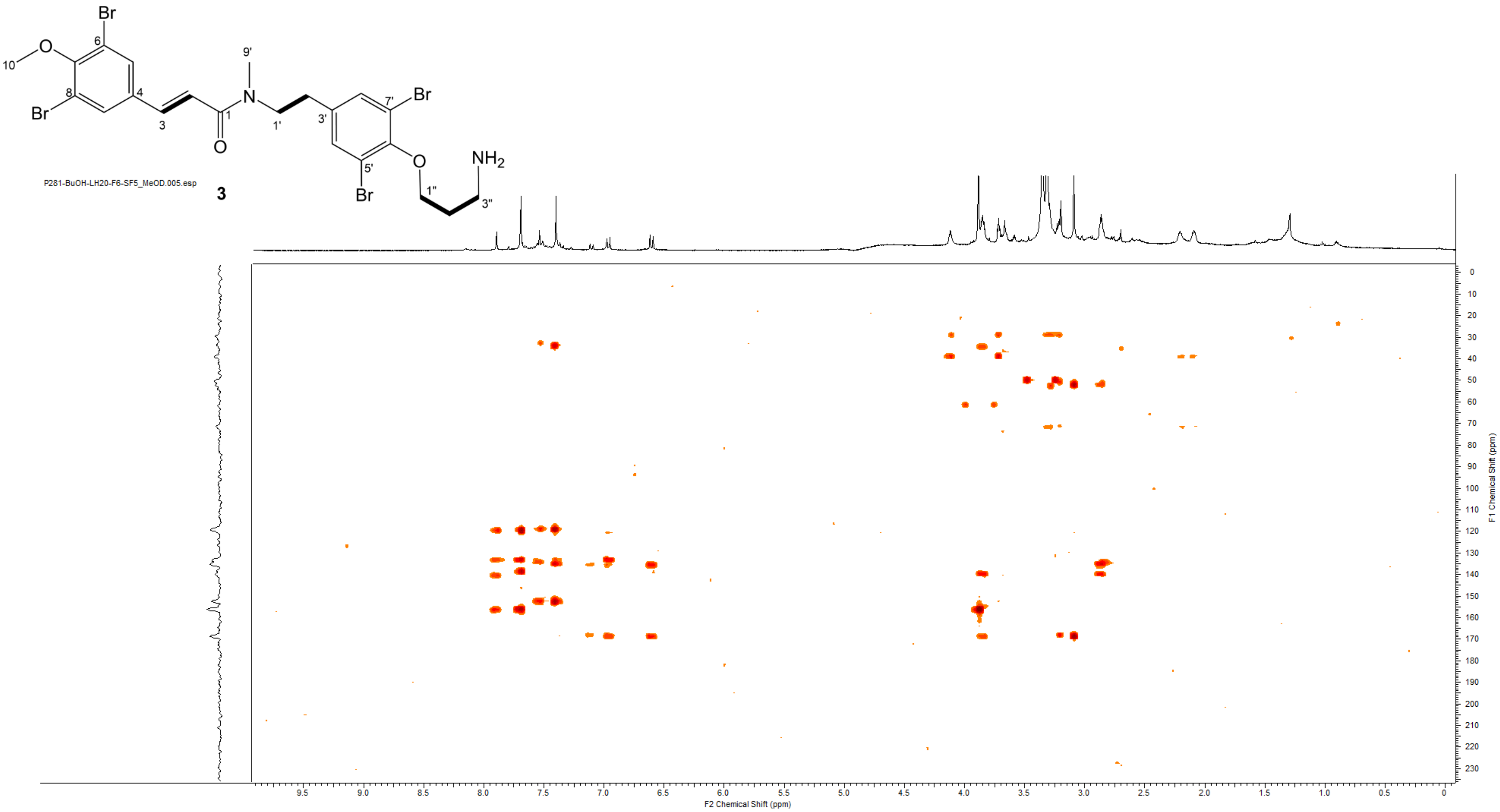

Figure S16. HR-ESI mass spectrum of Aplyzanzine E (**3**)

Elemental Composition Report **Page 1**

Single Mass Analysis
Tolerance = 10.0 mDa / DBE: min = -1.5, max = 50.0
Element prediction: Off
Number of isotope peaks used for i-FIT = 3

Monoisotopic Mass, Even Electron Ions
445 formula(e) evaluated with 12 results within limits (all results (up to 1000) for each mass)
Elements Used:
C: 0-100 H: 0-1000 N: 0-10 O: 0-10 79Br: 2-2 81Br: 2-2

ALMOURABIT_moriou51-4 20 (0.533) Cm (15:30) 1: TOF MS ES+ 2.15e+004

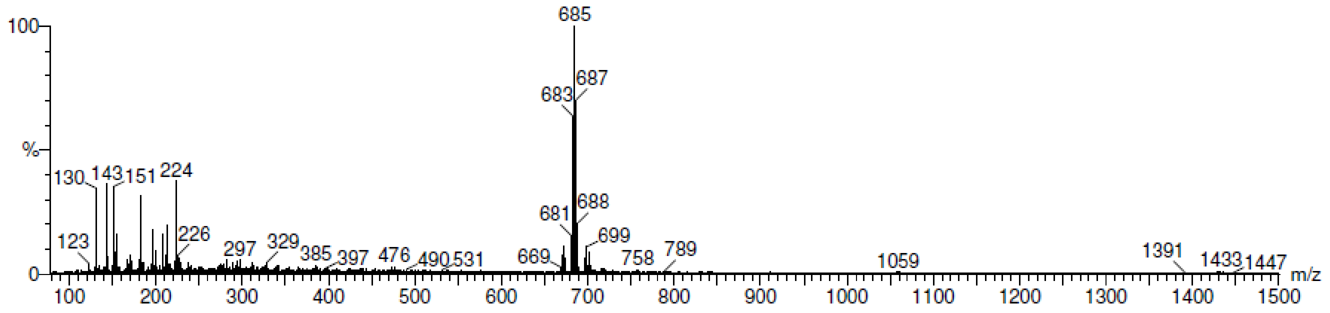

Minimum:				-1.5			
Maximum:		10.0	10.0	50.0			
Mass	Calc. Mass	mDa	PPM	DBE	i-FIT	i-FIT (Norm)	Formula
684.8616	684.8616	0.0	0.0	0.5	32.6	2.6	C15 H29 N2 O8 79Br2 81Br2
	684.8630	-1.4	-2.0	5.5	32.5	2.4	C16 H25 N6 O4 79Br2 81Br2
	684.8598	1.8	2.6	13.5	32.9	2.8	C27 H25 O 79Br2 81Br2
	684.8590	2.6	3.8	1.5	32.1	2.0	C11 H25 N8 O6 79Br2 81Br2
	684.8643	-2.7	-3.9	10.5	32.3	2.2	C17 H21 N10 79Br2 81Br2
	684.8657	-4.1	-6.0	4.5	32.9	2.8	C20 H29 O6 79Br2 81Br2
	684.8670	-5.4	-7.9	9.5	32.8	2.7	C21 H25 N4 O2 79Br2 81Br2
	684.8558	5.8	8.5	9.5	32.7	2.7	C22 H25 N2 O3 79Br2 81Br2

Figure S17. ^{1}H NMR spectrum of Aplyzanzine F (**4**) in MeOD (600 MHz)

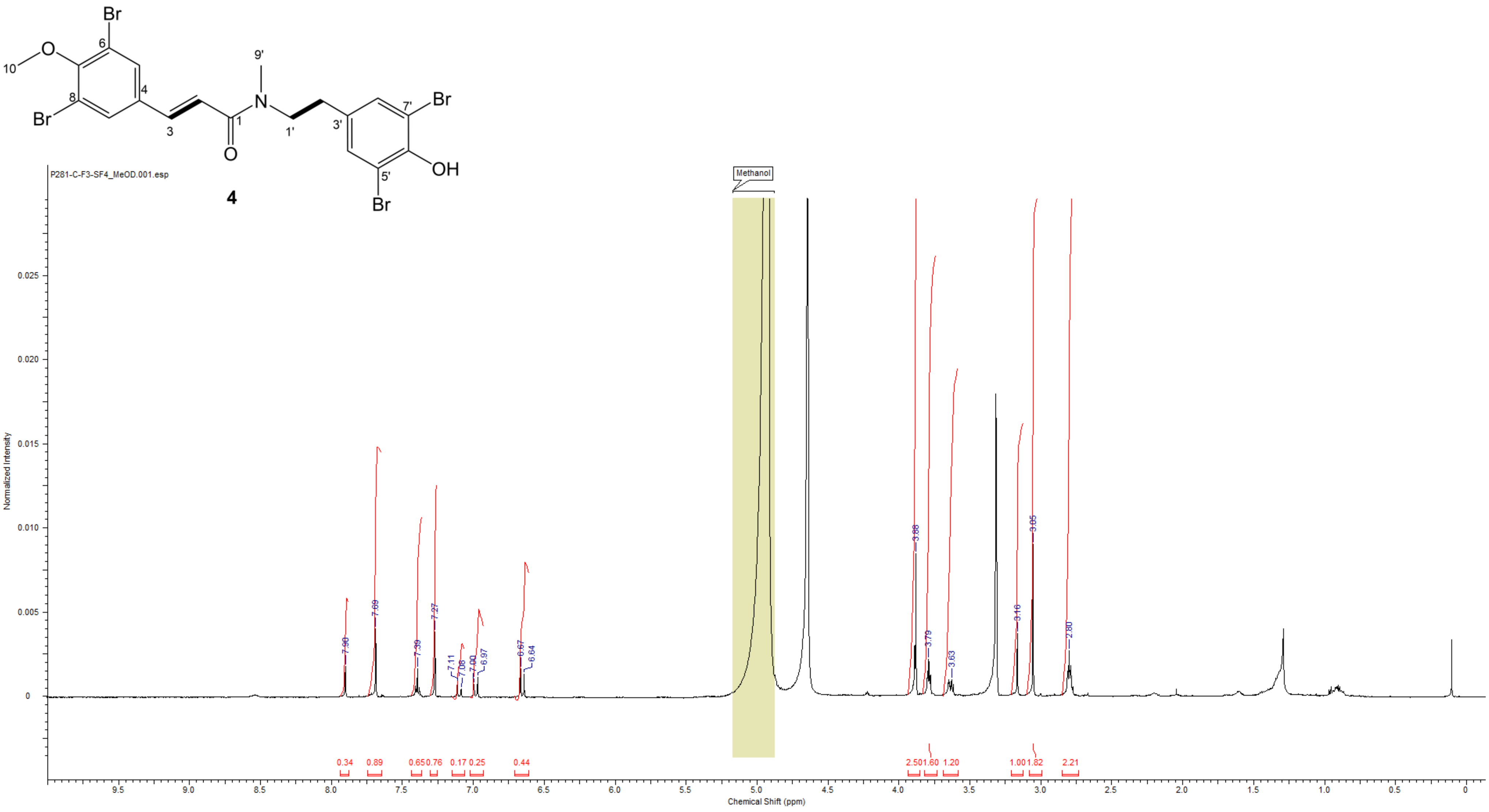

Figure S18. ^{1}H-^{1}H COSY NMR spectrum of Aplyzanzine F (**4**) in MeOD (600 MHz)

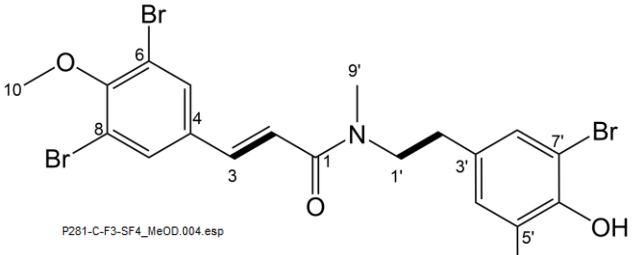

4

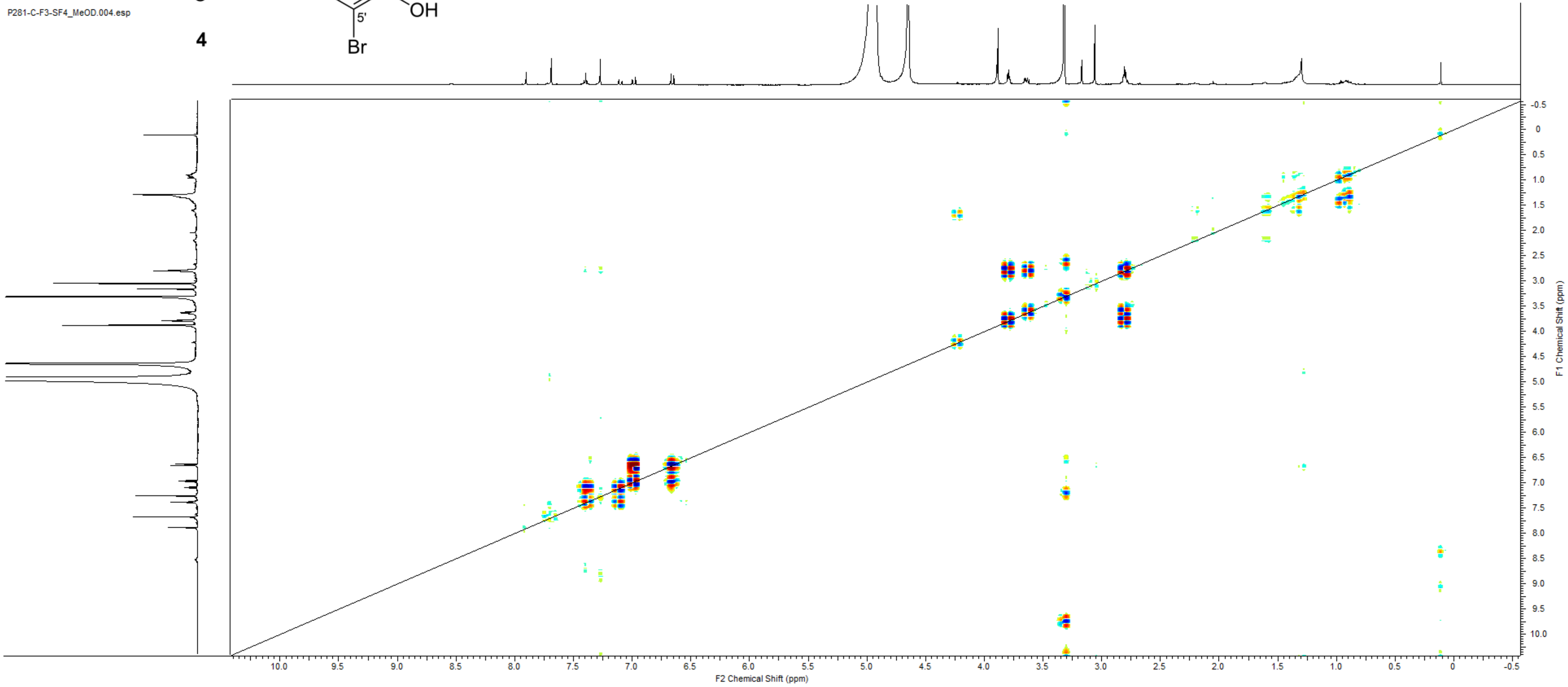

Figure S19. HSQC NMR spectrum of Aplyzanzine F (**4**) in MeOD (600 MHz)

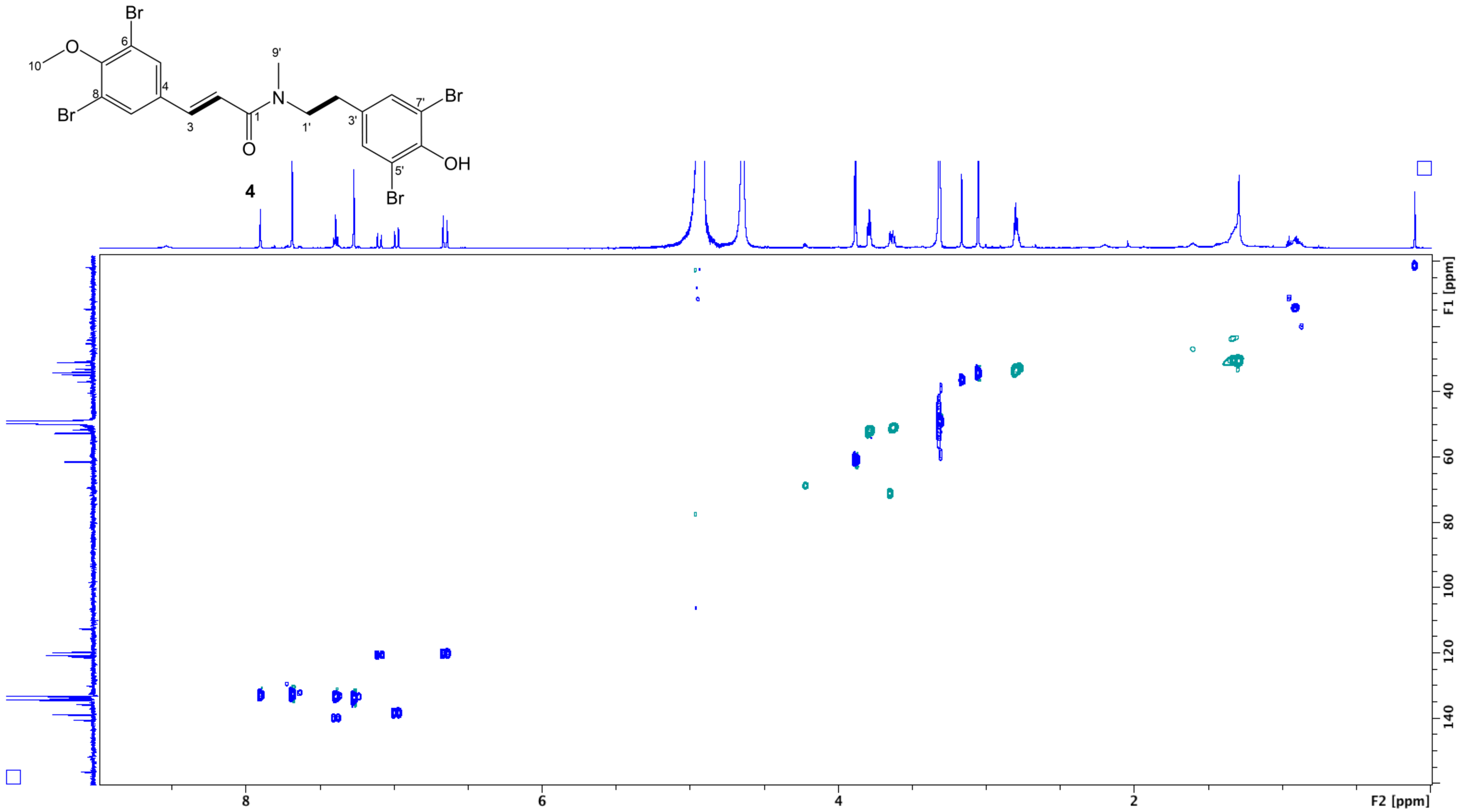

Figure S20. HMBC NMR spectrum of Aplyzanzine F (**4**) in MeOD (600 MHz)

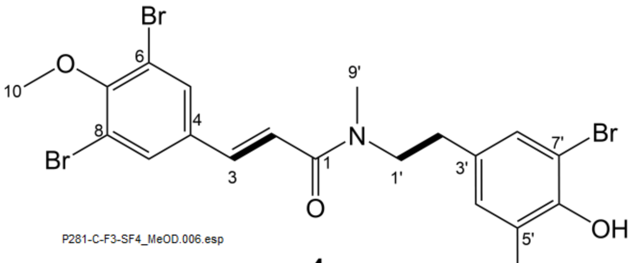

4

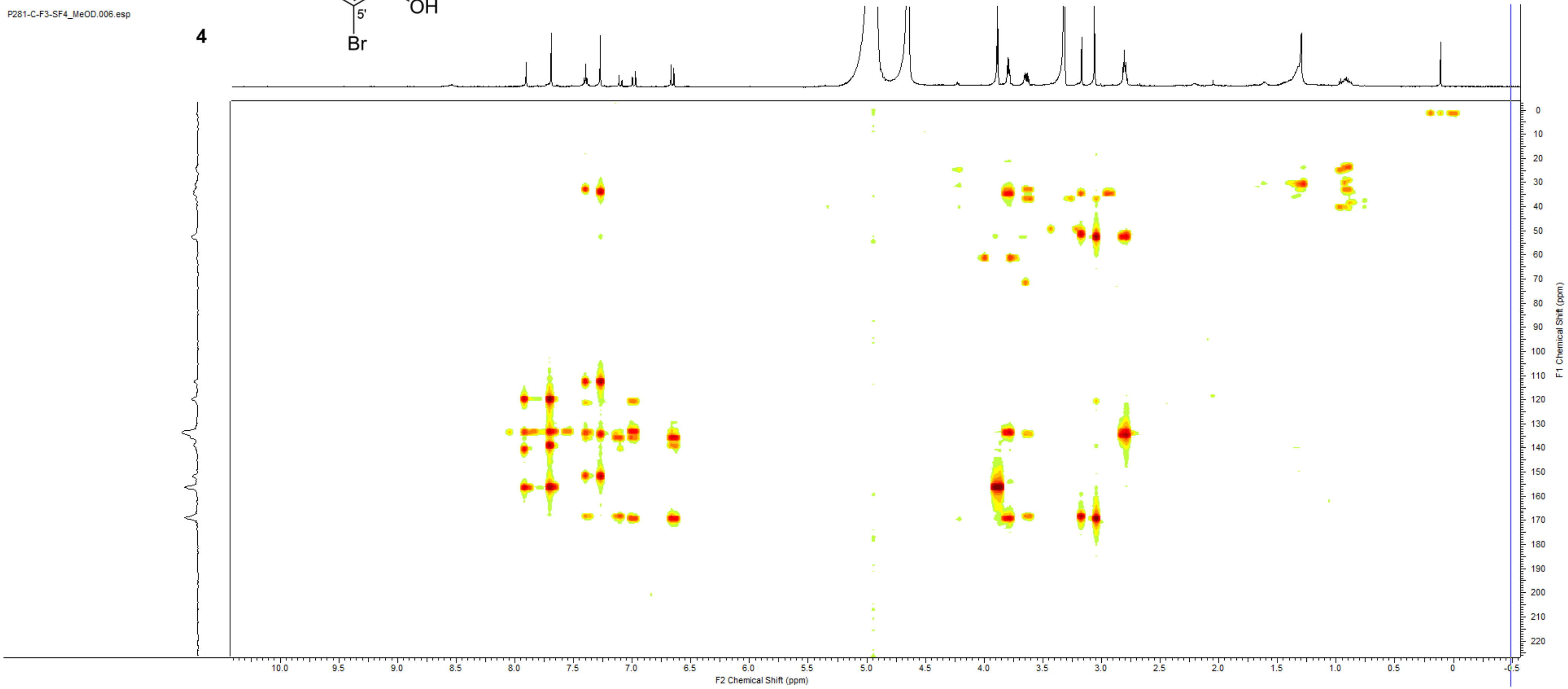

Figure S21. HR-ESI mass spectrum of Aplyzanzine F (**4**)

Elemental Composition Report

Single Mass Analysis

Tolerance = 50.0 mDa / DBE: min = -1.5, max = 50.0
Element prediction: Off
Number of isotope peaks used for i-FIT = 3

Monoisotopic Mass, Even Electron Ions
166 formula(e) evaluated with 28 results within limits (up to 50 closest results for each mass)
Elements Used:
C: 0-50 H: 0-100 N: 1-8 O: 2-8 79Br: 2-2 81Br: 2-2

ALMOURABIT_moriou32-2 22 (0.593) Cm (17:36)
1: TOF MS ES+
4.16e+004

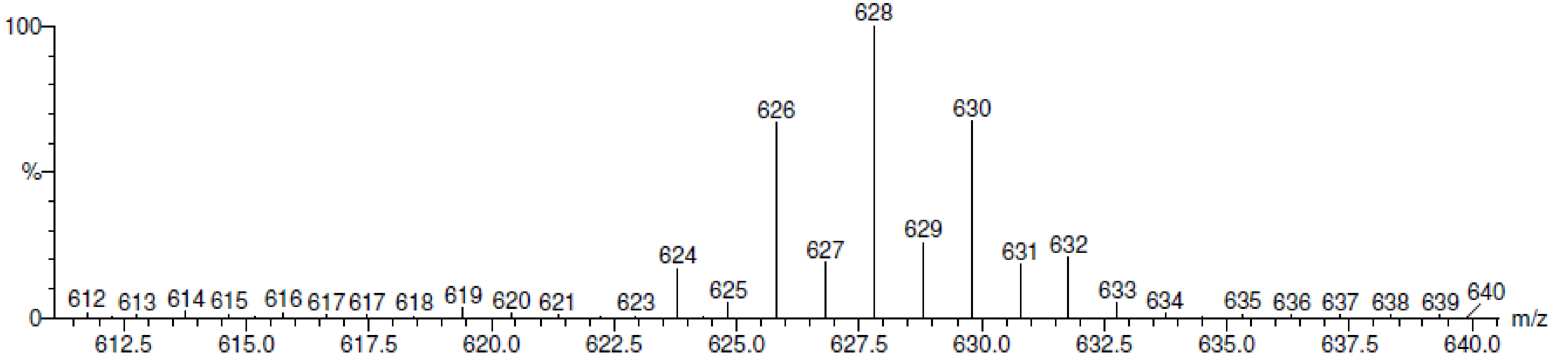

Minimum:				-1.5			
Maximum:		50.0	10.0	50.0			
Mass	Calc. Mass	mDa	PPM	DBE	i-FIT	i-FIT (Norm)	Formula
627.8020	627.8011	0.9	1.4	1.5	32.9	3.1	C8 H18 N7 O6 79Br2 81Br2
	627.8038	-1.8	-2.9	0.5	32.9	3.1	C12 H22 N O8 79Br2 81Br2
	627.8051	-3.1	-4.9	5.5	33.0	3.1	C13 H18 N5 O4 79Br2 81Br2
	627.7979	4.1	6.5	9.5	33.5	3.7	C19 H18 N O3 79Br2 81Br2

Figure S22. ^{1}H NMR spectrum of **5** in MeOD (300 MHz)

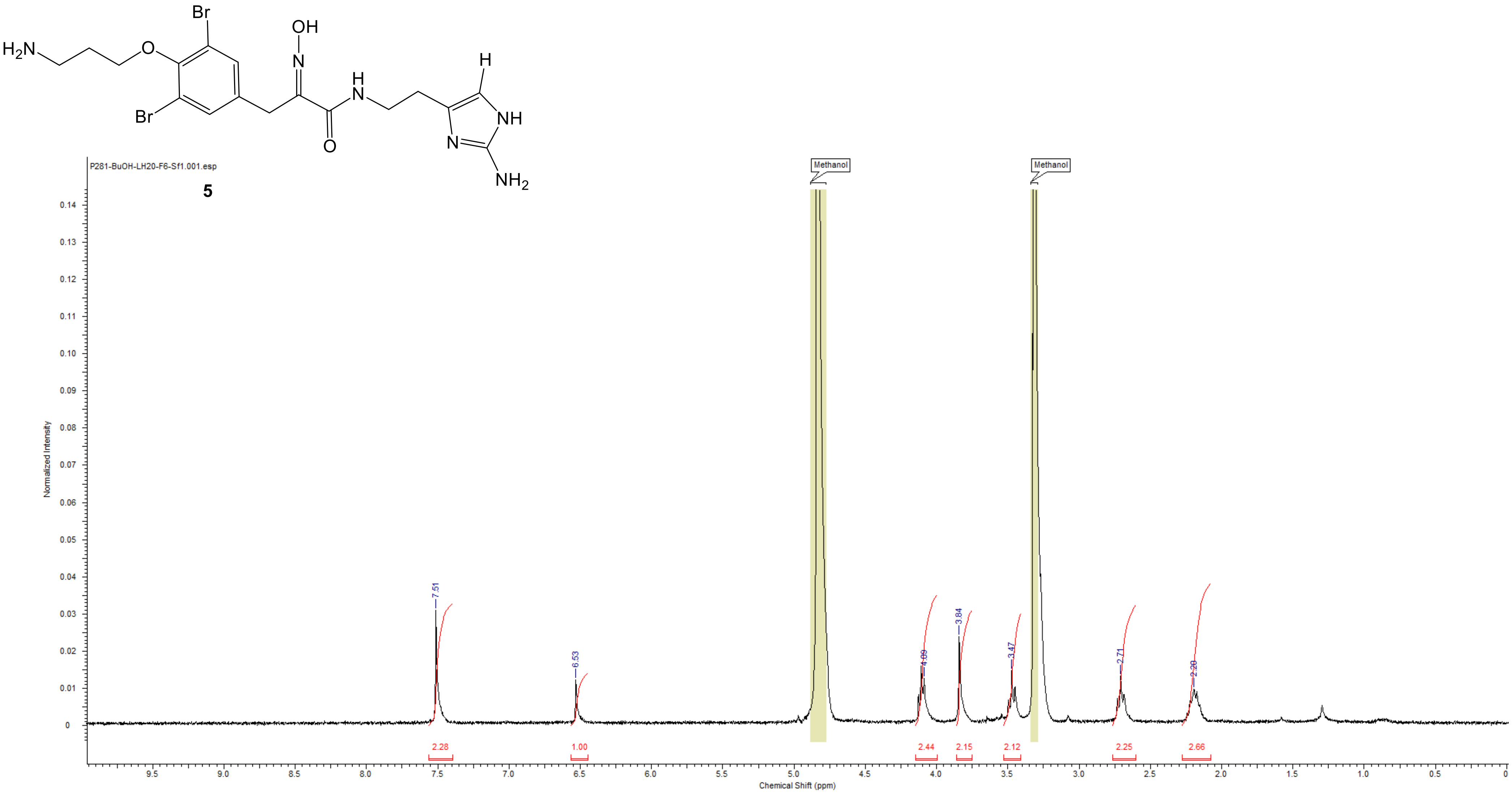

Figure S23. HR-ESI mass spectrum of **5**

Elemental Composition Report

Single Mass Analysis

Tolerance = 10.0 mDa / DBE: min = -1.5, max = 50.0
Element prediction: Off
Number of isotope peaks used for i-FIT = 3

Monoisotopic Mass, Even Electron Ions
452 formula(e) evaluated with 12 results within limits (all results (up to 1000) for each mass)
Elements Used:
C: 0-100 H: 0-1000 N: 0-10 O: 0-10 79Br: 1-1 81Br: 1-1

ALMOURABIT_moriou51-1 21 (0.572) Cm (16:31)

1: TOF MS ES+
1.39e+004

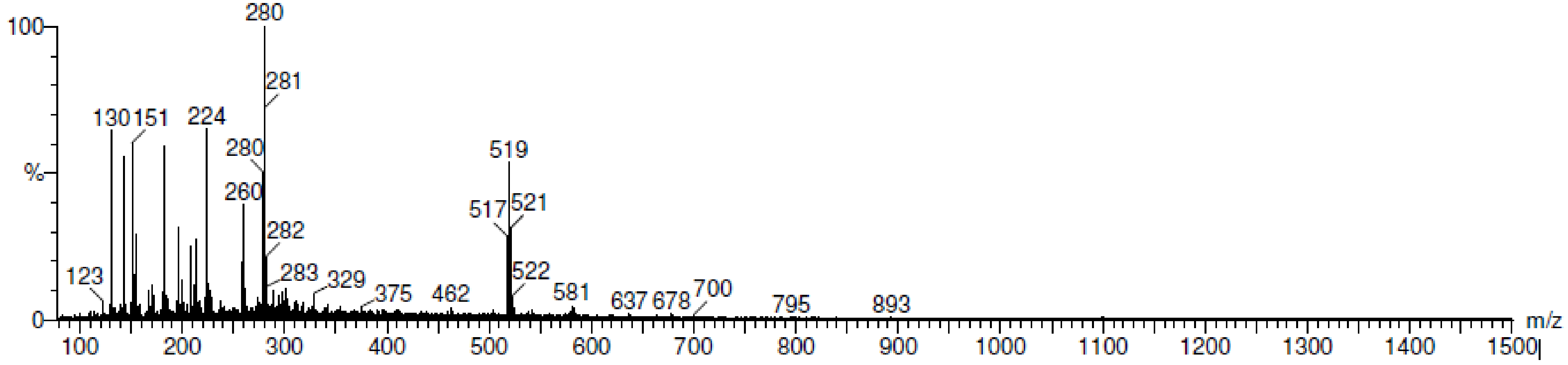

Minimum:				-1.5			
Maximum:		10.0	10.0	50.0			
Mass	Calc. Mass	mDa	PPM	DBE	i-FIT	i-FIT (Norm)	Formula
519.0208	519.0205	0.3	0.6	7.5	29.7	2.9	C21 H27 O5 79Br 81Br
	519.0218	-1.0	-1.9	12.5	29.7	2.9	C22 H23 N4 O 79Br 81Br
	519.0237	-2.9	-5.6	-0.5	29.0	2.1	C10 H27 N6 O8 79Br 81Br
	519.0178	3.0	5.8	8.5	29.3	2.5	C17 H23 N6 O3 79Br 81Br

Table S1. Comparison of the spectroscopic data of the compound **5** with the data from the literature concerning the purealidin A[54] and the lipopurealin D[56].

Compound **5**	Purealidin A
MS data	
HRESIMS data showed the $[M+H]^+$ ion as a cluster at *m/z* 517, 519, 521, in a 1:2:1 ratio, indicating the presence of two bromine atoms in the molecule. Analysis of the pic $[M+H]^+$ at *m/z* 519.0208 (*m/z* 519.0178 calcd. for $C_{17}H_{23}N_6O_3{}^{79}Br^{81}Br$, Δ= 5.8ppm) allowed us to propose the molecular formula of **5** as $C_{17}H_{22}N_6O_3Br_2$.	FABMS (positive ion, glycerol matrix) *m/z* 559, 557, 555 [ca. 1:2:1, $(M + K)^+$], 521,519, 517 [ca. 1:2:1, $(M + H)^+$], 505, 503,501 [ca. 1:2:1, $(M + H\text{-}NH_2)^+$], 441, and 439 [ca. 1:1, $(M\text{-}Br)^+$]; HRFABMS (positive) found *m/z* 517.0241, calcd for $C_{17}H_{23}N_6O_3{}^{79}Br_2$ (M + H) 517.0198.

1D and 2D NMR data					
	in CD_3OD		in DMSO-*d6*		
Position	Lipopurealin D[56]	**5**	Purealidin A[54]		
No	δ H mult, (*J* in Hz)	δ H mult	δ C, type	δ H mult, (*J* in Hz)	^1H-^1H COSY
1	3.43, t (7)	3.31, m	36.5, CH_2	3.06, m	NH_2-1, 2
NH_2-1				7.83, br s	1
2	2.03, m	2.20, m	27.9, CH_2	2.02, m	1, 3
3	4.00, t (6)	4.09, m	70.4, CH_2	3.98, t (6)	2
4			150.5, C		
5, 5′			117.1, C		
6, 6′	7.46, s	7.51, s	132.9, CH	7.45, s	8
7			136.4, C		
8	3.82, s	3.84, s	27.7, CH_2	3.75, s	6, 6′
9			150.9, C		
NOH-9				12.03, s	
10			163.1, C		
NH-10				8.15, t (6)	11
11	3.45, t (7)	3.45, m	37.3, CH_2	3.36, dt (6)	NH-10, 12
12	2.66, t (7)	2.71, m	24.4, CH_2	2.59, t (6)	11
13			124.3, C		
NH-13				11.77, br s	14
14	6.41, s	6.53, s	109.2, CH	6.57, s	NH-13, NH-14
NH-14				12.16, br s	14
15			146.9, C		

Figure S24: Lipopurealin D[56] and general key COSY for purealidin A in DMSO-*d6*.[54]

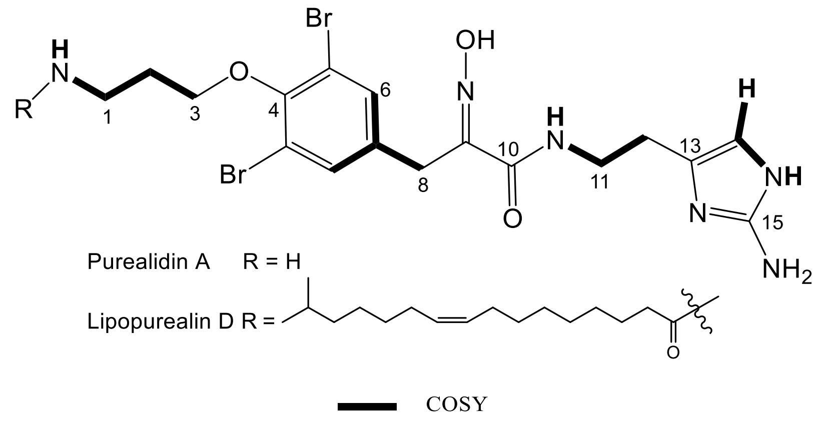

Figure S25. ^{1}H NMR spectrum of **6** in MeOD (500 MHz)

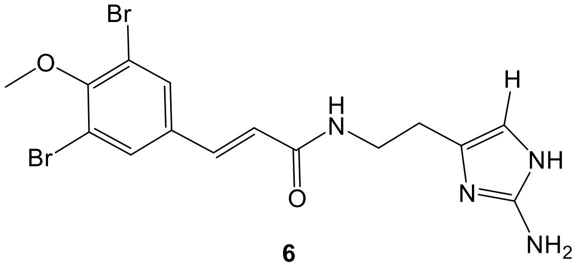

6

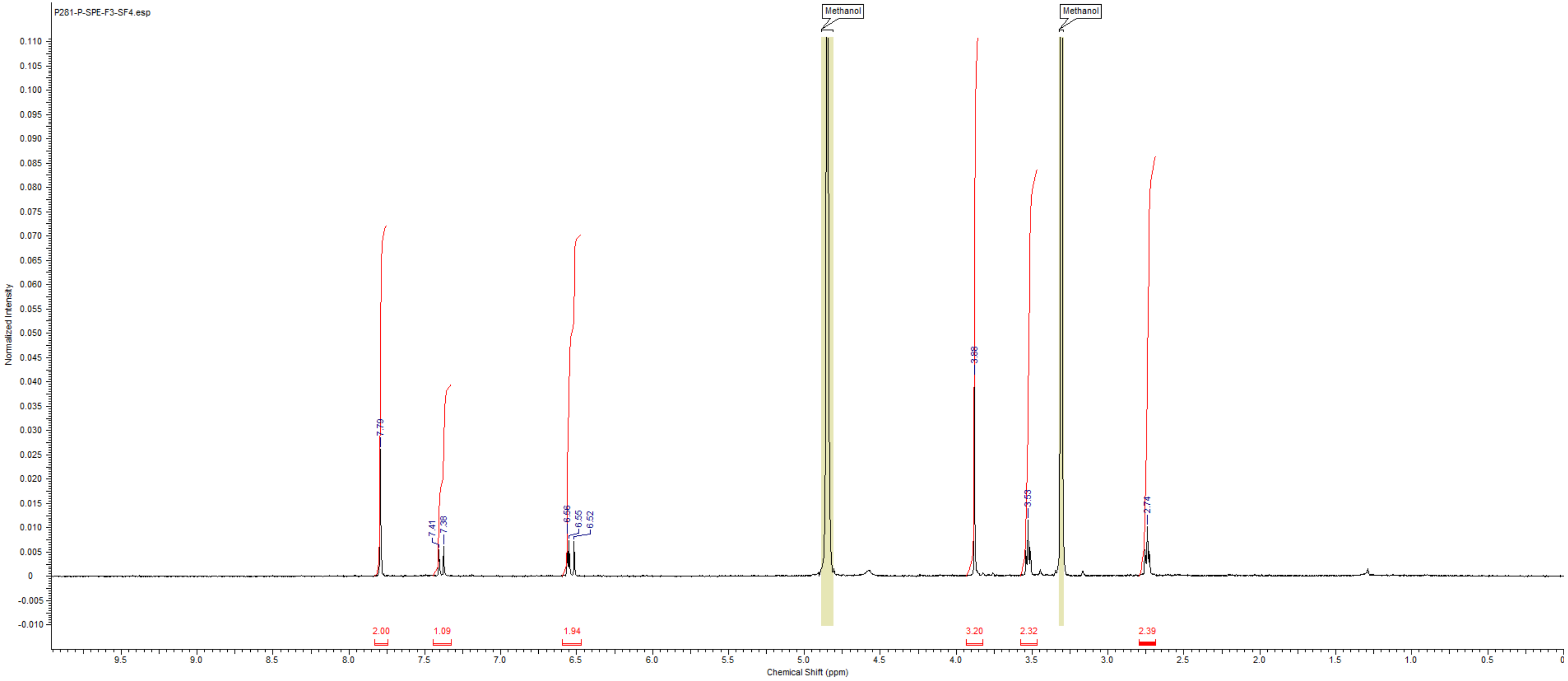

Figure S26. HR-ESI mass spectrum of **6**

Elemental Composition Report

Single Mass Analysis

Tolerance = 10.0 mDa / DBE: min = -1.5, max = 50.0
Element prediction: Off
Number of isotope peaks used for i-FIT = 3

Monoisotopic Mass, Even Electron Ions
339 formula(e) evaluated with 11 results within limits (all results (up to 1000) for each mass)
Elements Used:
C: 0-100 H: 0-1000 N: 0-10 O: 0-10 79Br: 1-1 81Br: 1-1

ALMOURABIT_moriou51-3 21 (0.571) Cm (16:28)

1: TOF MS ES+
1.12e+004

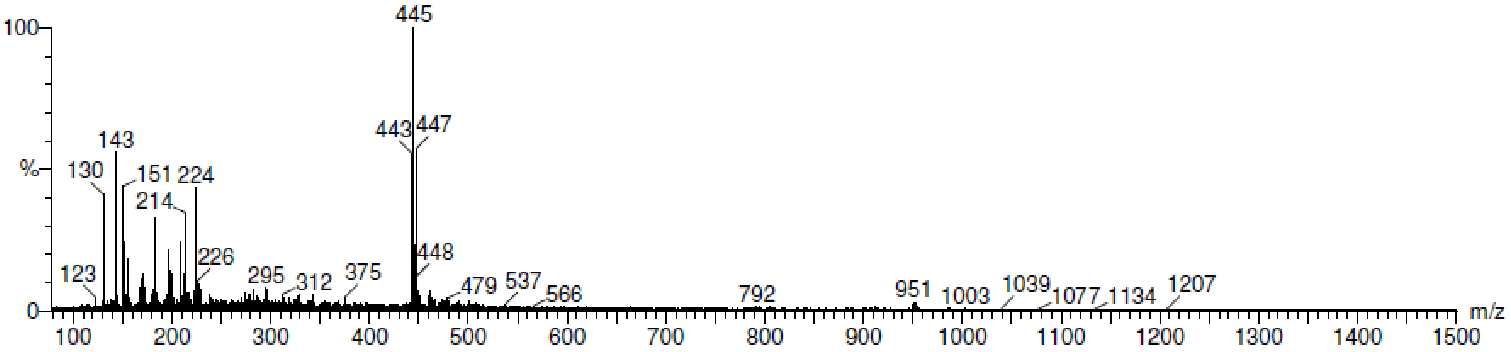

Minimum:				-1.5			
Maximum:		10.0	10.0	50.0			
Mass	Calc. Mass	mDa	PPM	DBE	i-FIT	i-FIT (Norm)	Formula
444.9745	444.9738	0.7	1.6	12.5	37.5	2.5	C20 H17 N2 79Br 81Br
	444.9757	-1.2	-2.7	-0.5	37.4	2.4	C8 H21 N4 O7 79Br 81Br
	444.9730	1.5	3.4	0.5	37.7	2.7	C4 H17 N10 O5 79Br 81Br
	444.9770	-2.5	-5.6	4.5	37.4	2.4	C9 H17 N8 O3 79Br 81Br
	444.9698	4.7	10.6	8.5	37.4	2.4	C15 H17 N4 O2 79Br 81Br

Table S2. Comparison of the spectroscopic data of the compound **6** with the data from the literature.[55]

Position No	Compound **6**	Compound 11 in Ciminiello et al.[55]	
MS data			
	HRESIMS data showed the $[M+H]^+$ ion as a cluster at *m/z* 443, 445, 447, in a 1:2:1 ratio, indicating the presence of two bromine atoms in the molecule. Analysis of the pic $[M+H]^+$ at *m/z* 444.9745 (*m/z* 444.9698 calcd. for $C_{15}H_{17}N_4O_2{}^{79}Br^{81}Br$, Δ= 10.6ppm) allowed us to propose the neutral molecular formula of **6** as $C_{15}H_{16}N_4O_2Br_2$.	Compound 11 showed intense M+ ions in the ratio of about 1:2:1 at m/z 443, 445, 447 in the positive fabms, which indicated the presence of two bromine atoms in the molecule. Combined analysis of the low-resolution ms and the 1H and 13C nmr spectra indicated a molecular formula of $C_{15}H_{17}N_4O_2Br_2$ for compound 11.	
1D NMR data in CD_3OD			
	δ_H mult, (*J* in Hz)	δ_C	δ_H mult, (*J* in Hz)
1		136.4	
2, 6	7.79, s	133.0	7.82, s
3, 5		119.5	
4		154.5	
7	7.40, d (15.5)	138.4	7.42, d (15.5)
8	6.53, d (15.5)	123.9	6.57, d (15.5)
9		167.5	
10	3.53, t	39.3	3.56, t (7)
11	2.74, t	25.9	2.77, t (7)
12		125.9	
13	6.56, s	110.9	6.59, s
14		146.4	
15	3.88, s	61.3	3.91, s

Figure S27: Structure of the compound 11 in Ciminiello et al.[55]

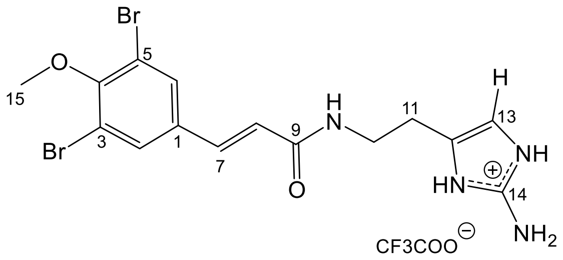

Supplement: Supplementary file 1 [file marinedrugs-18-00272-s001.pdf]
